# Efficacy and safety of anti-angiogenic drugs combined with chemotherapy in the treatment of platinum-sensitive/resistant ovarian cancer: a meta-analysis with trial sequential analysis of randomized controlled trials

**DOI:** 10.3389/fphar.2024.1446403

**Published:** 2024-11-21

**Authors:** Haining He, Fei Zhou

**Affiliations:** Department of Obstetrics and Gynaecology, Sichuan Provincial People’s Hospital, School of Medicine, University of Electronic Science and Technology of China, Chengdu, China

**Keywords:** anti-angiogenic, VEGFR, chemotherapy, bevacizumab, platinum-sensitive, platinum-resistant, ovarian cancer, meta-analysis

## Abstract

**Background:**

With the emergence of new anti-angiogenic treatments and the ongoing updates to clinical guidelines, the effectiveness and safety of these agents in treating platinum-sensitive/resistant ovarian cancer (OC) are yet to be fully determined. Therefore, we conducted a meta-analysis to evaluate the efficacy and safety of anti-angiogenic drugs combined with chemotherapy (CT) for platinum-sensitive OC (PSOC) or platinum-resistant OC (PROC).

**Methods:**

A comprehensive literature search was conducted across several databases, including PubMed, Web of Science, Embase, and the Cochrane Library, encompassing all pertinent randomized controlled trials (RCTs) up to 31 May 2024. The primary outcomes for the meta-analysis were progression-free survival (PFS) and overall survival (OS), while the objective response rate (ORR), adverse events (AEs) of any grade, and grade ≥3 AEs were considered secondary endpoints. Data synthesis involved the computation of hazard ratio (HR), relative risk (RR), along with their 95% confidence interval (CI) and prediction interval (PI). Trial sequential analysis was carried out using STATA 12.0, R software 4.3.1, and TSA v0.9.5.10 Beta software.

**Results:**

This meta-analysis encompassed 15 RCTs. The overall analysis revealed that compared to CT alone (or plus placebo), anti-angiogenic drugs combined with CT significantly improved PFS (HR [95% CI] = 0.573 [0.518–0.633], 95% PI: 0.383-0.876) and ORR (RR [95% CI] = 1.362 [1.260–1.472], 95% PI: 0.824–2.251), but also increased the incidence of grade ≥3 AEs (RR [95% CI] = 1.115 [1.070–1.162], 95% PI: 0.870–1.422) in PSOC patients. For PROC patients, this combination therapy notably improved PFS (HR [95% CI] = 0.542 [0.475–0.619], 95% PI: 0.322–0.930), OS (HR [95% CI] = 0.752 [0.646–0.875], 95% PI: 0.554-0.997), and ORR (RR [95% CI] = 2.141 [1.702–2.694], 95% PI: 0.839–5.307), whilst simultaneously elevating the risk of grade ≥3 AEs (RR [95% CI] = 1.487 [1.216–1.819], 95% PI: 0.755–2.828).

**Conclusion:**

Our research verified the advantages of combining anti-angiogenic agents with CT in enhancing PFS and ORR for patients with PSOC, and also confirmed improvements in PFS, OS, and ORR for those with PROC. It is crucial for medical practitioners to remain alert to the potential occurrence of AEs when implementing this combined therapeutic approach in a clinical milieu.

**Systematic Review Registration:**

https://www.crd.york.ac.uk/PROSPERO/, identifier CRD42024552010.

## 1 Introduction

Among all gynecological malignancies, ovarian cancer (OC) stands as the most lethal, ranking as the eighth leading cause of cancer among females globally (Siegel et al., 2022). Initial treatment strategies typically involve surgical procedures and a regimen of platinum-based chemotherapy (CT). Despite a majority of patients initially responding to a combined regimen of platinum and taxane, a disheartening 75%–80% of those diagnosed with advanced stages of the disease will face a relapse within a year and a half (Webber and Friedlander, 2017). Platinum-sensitive OC (PSOC) is defined as a tumor that initially achieves complete remission and then relapse at least 6 months later (partially platinum-sensitive) or after 12 months (platinum-sensitive) following previous CT (Colombo et al., 2019). In contrast, patients with a platinum-free interval (PFI) < 6 months (or PFI < 1 month or progression during first-line therapy) are classified as having platinum-resistant OC (PROC) (or platinum-refractory OC) (Newhouse et al., 2023). In these cases, re-administration of platinum-based CT can result in response rates ranging from 30% to 90%, and median survival can extend up to 45 months (Davis et al., 2014; Nishio et al., 2009; Rose et al., 2019). Relapsed OC is broadly deemed incurable, with the malignancy progressively developing resistance to CT (Matulonis et al., 2016). Consequently, the quest for alternative therapeutic strategies for women grappling with relapsed OC remains a pressing and unfulfilled requirement.

In the field of OC research, a key strategy involves the integration of CT and targeted therapies to augment their impact, or the application of these drugs as maintenance treatments to extend the duration to disease progression and subsequent treatment, ultimately aiming to enhance survival rates. A method is the suppression of angiogenesis, the mechanism behind the creation of new blood vessels vital for tumor expansion (Hicklin and Ellis, 2005). Angiogenesis is integral to both typical ovarian physiology and the advancement of OC, contributing to ascites formation and metastatic distribution. Its inhibition has demonstrated potential clinical advantages for OC patients, inclusive of those with platinum-sensitive/resistant conditions (Monk et al., 2016a; Mullen et al., 2019). Anti-angiogenic drugs are emerging as a hopeful category of treatments for patients with PSOC or PROC. Evidence from the AURELIA trial revealed significant benefits of adding bevacizumab, a vascular endothelial growth factor (VEGF) inhibitor, to cytotoxic CT (comprising topotecan, pegylated liposomal doxorubicin, or weekly paclitaxel). This combination extended progression-free survival (PFS) by 3.3 months and increased the objective response rate (ORR) by 15.5% in PROC patients who had undergone a maximum of two prior treatments. Regrettably, this trial failed to provide an overall survival (OS) benefit (Pujade-Lauraine et al., 2014). The ICON6 trial provided further evidence, demonstrating that CT in conjunction with cediranib, an oral VEGF receptor (VEGFR) inhibitor, significantly enhanced PFS in PSOC patients. However, the improvement in OS was not statistically significant when compared to the placebo group (Ledermann et al., 2021; Ledermann et al., 2016).

Over the past several years, a variety of randomized controlled trials (RCTs) have been conducted to evaluate the clinical efficacy of anti-angiogenic drugs combined with CT and CT alone for treating PSOC or PROC. However, these studies have reported variable findings (Duska et al., 2020; Hall et al., 2020; Pignata et al., 2021; Wang et al., 2022). Moreover, whether the addition of angiogenesis inhibitors to CT results in an increased risk of adverse events (AEs) remains controversial (Aghajanian et al., 2015; Chekerov et al., 2018). Therefore, we performed a meta-analysis to compare the efficacy and safety of anti-angiogenic agents in combination with CT *versus* CT alone (or plus placebo) in the treatment of PSOC or PROC.

## 2 Materials and methods

### 2.1 Study design

In accordance with the Preferred Reporting Items for Systematic Reviews and Meta-Analyses (PRISMA) guidelines (Page et al., 2021), the approach and presentation of our study were developed. Our study protocol, moreover, has been registered in the PROSPERO database, under the registration number: CRD42024552010. As our research is a meta-analysis that compiles results from previously published studies, it is exempt from requiring ethical approval and informed consent because it does not involve ethical concerns or infringe upon patient privacy.

### 2.2 Literature search strategy

We conducted an exhaustive search across several databases, including PubMed, Web of Science, Embase, and the Cochrane Library of clinical trials, with the objective of locating all pertinent articles published in English up to 31 May 2024. The primary search terms we utilized encompassed: (“bevacizumab”, “pazopanib”, “trebananib”, “nintedanib”, “cediranib”, “sorafenib”, “perifosine”, “votrient”, “recentin”, “afibercept”, “anlotinib”, “apatinib”) OR (“antiangiogenetic”, “anti-angiogenic”, “anti-angiogenesis”, “angiogenesis inhibitor”, “vascular endothelial growth factor”, “anti-VEGF”, “VEGF”, “VEGFR”) AND (“cancer*“, “carcinoma*“, “malignan*“, “neoplasm*“, “tumour*“, “tumor*“) AND “ovar*“). An updated literature search was conducted on 25 October 2024. The detailed outline of our search strategy is available in Supplementary File S1. Additionally, we conducted a manual examination of references cited within relevant review articles to unearth any further studies potentially satisfying our eligibility criteria.

### 2.3 Inclusion and exclusion criteria

The selection of studies for inclusion was based on specific criteria: (i) Only RCTs were considered; (ii) Study participants were female adult subjects (18 years or older) with a diagnosis of PSOC or PROC confirmed by histological analysis; (iii) The intervention involved the use of anti-angiogenic agents in combination with CT; (iv) The control condition was CT alone or with placebo; (v) The outcomes included PFS, OS, ORR, any grade adverse events (AEs) or grade ≥3 AEs. Exclusion criteria were applied to (i) retrospective, non-interventional, non-comparative, or single-arm studies; (ii) studies that did not report relevant outcomes or contained duplicated data; (iii) designs where both the intervention and control cohorts were treated with anti-angiogenic agents; (iv) case reports, commentaries, literature reviews, study protocols, and conference abstracts.

### 2.4 Data extraction and quality assessment

The screening, selection, exclusion, and data extraction processes were carried out by two separate evaluators. For each RCT included, we gathered information including the first author’s name, publication year, study phase, trial name, patient population, participant count and median age, the specific anti-angiogenic drugs used, dosage and treatment cycles for the experimental and control arms, the follow-up duration, and the outcomes. The primary outcomes of interest for the meta-analysis were PFS and OS, while the ORR, AEs of any grade, and grade ≥3 AEs were considered secondary endpoints. In instances of multiple publications pertaining to a single RCT, the most recent or comprehensive report was prioritized to ensure the inclusion of complete and current data. Where direct reports of PFS or OS were unavailable, we utilized the Engauge Digitizer Version 10.8 software (http://markummitchell.github.io/engauge-digitizer/) alongside the method proposed by Tierney and colleagues (Tierney et al., 2007) to estimate the data from Kaplan-Meier curves (Xie et al., 2022).

The assessment of the RCTs’ quality was carried out using the modified Jadad scale (Jadad et al., 1996), a tool encompassing criteria like the randomization procedure, the concealment of this randomization, the application of double-blind techniques, and the documentation of participant withdrawals and discontinuations. The categorization of trials was predicated on their quality scores: a range of 0–3 was indicative of low quality, whereas a score within the 4 to 7 bracket was representative of high-quality research.

### 2.5 Statistical analysis

This study quantified aggregate hazard ratios (HRs) with corresponding 95% confidence intervals (CIs) for PFS and OS. For dichotomous outcomes, relative risks (RRs) were calculated and accompanied by 95% CIs. Heterogeneity among the included studies was evaluated through I^2^ statistics, Cochran’s Q test, and the application of the 95% prediction interval (PI) (Bowden et al., 2011; IntHout et al., 2016). Instances where I^2^ exceeded 50% or the *p*-value was below 0.10 were considered to exhibit significant heterogeneity, leading to the adoption of a random-effects model; otherwise, a fixed-effects model was applied (Higgins and Thompson, 2002). Subgroup analyses were conducted to explore differences across the categories of anti-angiogenic agents used. Sensitivity analysis was undertaken to pinpoint potential sources of heterogeneity. Additionally, funnel plots and Begg’s and Egger’s tests were utilized for identifying any publication bias (Begg and Mazumdar, 1994; Egger et al., 1997). All data analyses were conducted utilizing R software 4.3.1 and STATA Version 12.0. Statistical significance was established at a two-sided *p*-value of less than 0.05.

### 2.6 Trial sequential analysis

A trial sequential analysis (TSA) was performed to ascertain if the aggregated data reached the required information size (RIS) for definitive conclusions (Wetterslev et al., 2017). This analysis, pertinent to dichotomous outcomes, was facilitated through the use of TSA software v0.9.5.10 Beta (www.ctu.dk/tsa), where the RIS was determined, and O’Brien-Fleming α-spending boundaries were set, adhering to a 5% type I error rate and a 20% type II error rate for bilateral tests. We utilized STATA Version 12.0, implementing the “metacumbounds” and “rsource” function, and R software 4.3.1, employing the “foreign” and “ldbounds” packages, to carry out TSA on the PFS and OS data, using the *a priori* information size (APIS) approach. The event of the cumulative Z-curve surpassing the RIS (or APIS) demarcation or the trial sequential monitoring boundary was interpreted as a signal that no additional studies are required, and the evidence could be deemed definitive.

## 3 Results

### 3.1 Study selection

**FIGURE 1:**
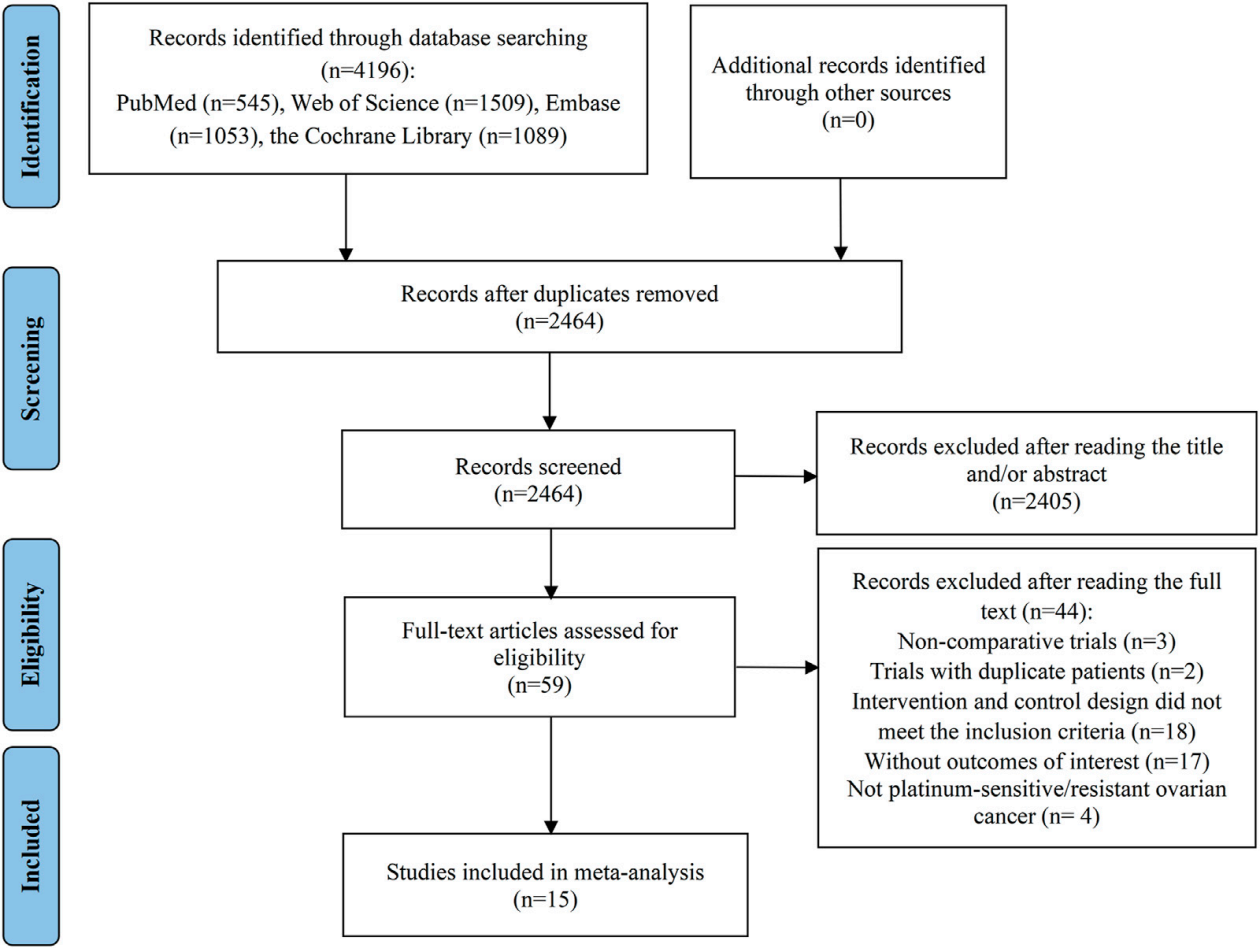
Flow diagram of the process of study selection.

The initial database query produced a total of 4,196 records. After the elimination of 1732 duplicate entries, 2,464 records remained for further assessment. Of these, 2,405 were deemed irrelevant based on their titles or abstracts, leaving a subset of 59 articles for a comprehensive full-text review to assess their suitability for inclusion. Following a thorough evaluation, 44 studies were found inappropriate for inclusion: 3 studies were non-comparative in nature; 2 trials contained repeated patient data; 18 trials had intervention and control schemes that did not meet the inclusion standards; 17 articles did not present the required outcome data; and 4 studies included participants who were not diagnosed with PSOC or PROC. Ultimately, 15 RCTs were chosen for inclusion in the meta-analysis (Aghajanian et al., 2012; Aghajanian et al., 2015; Chekerov et al., 2018; Coleman et al., 2017; Duska et al., 2020; Hall et al., 2020; Ledermann et al., 2021; Ledermann et al., 2016; Pignata et al., 2021; Pignata et al., 2015; Pujade-Lauraine et al., 2014; Richardson et al., 2018; Roque et al., 2022; Shoji et al., 2022; Wang et al., 2022) (Figure 1).

### 3.2 Characteristics and quality assessment of included studies

Table 1 presented a comprehensive breakdown of the features of RCTs and the participants included in the analysis. Our study comprised 15 RCTs in total, with 8 being phase II trials and 7 being phase III trials. Among these, 8 trials were dedicated to PSOC patients, while 9 trials catered to PROC patients. Notably, the research conducted by Richardson et al. (2018) and Duska et al. (2020) documented results for both PSOC and PROC patients. The subject pool was made up of 2008 OC patients who were allocated to the group receiving anti-angiogenic agents in conjunction with CT, and 1909 OC patients who received CT either alone or with a placebo. The anti-angiogenic medications employed were divided into VEGF inhibitors (including bevacizumab only), and VEGFR inhibitors, which encompassed pazopanib, sorafenib, apatinib, cediranib, and nintedanib. Among the 15 RCTs, 13 were evaluated as high quality, while 2 were considered of low quality. A noteworthy methodological shortcoming identified in several RCTs was the absence of double-blinding within the trial designs (Supplementary File S2).

**TABLE 1:** The basic characteristics of the included RCTs.

Author (Year)	Study phase	Trial name	Patient population	Sample size (E/C)	Median age (E/C, years)	Types of anti-angiogenic drugs	Experimental arm	Control arm	Median follow-up duration (E/C, months)	Outcomes
Coleman et al. (2017)	Phase III	GOG-0213	Recurrent, platinum-sensitive, epithelial ovarian, primary peritoneal, or fallopian tube cancer; GOG PS of 0–2	337/337	59.5/60.6	VEGF inhibitor	Pac (175 mg/m^2^) + Car (AUC 5) + Bev (15 mg/kg), q3w	Pac (175 mg/m^2^) + Car (AUC 5), q3w	49.6	1, 2, 3, 5
Pignata et al. (2021)	Phase III	MITO16b/MANGO–OV2/ENGOT–ov17	Platinum-sensitive, FIGO stage IIIB-IV ovarian cancer, fallopian tube carcinoma, or peritoneal carcinoma; ECOG PS of 0–2	203/203	61/60	VEGF inhibitor	Car-based doublet + Bev (10 mg/kg intravenous every 14 days)	Car-based doublet, i.v	20.1	1, 2, 3, 5
Richardson et al. (2018)	Phase II	NCT01468909	Recurrent or persistent epithelial ovarian, fallopian tube, or primary peritoneal cancer; GOG PS of 0–1	54/52	61/61	VEGFR inhibitor	Pac (80 mg/m^2^ on days 1, 8 and 15 every 28 days) + Paz 800 mg daily	Pac (80 mg/m^2^ on days 1, 8 and 15 every 28 days) + PL 800 mg daily	17.7	1
Duska et al. (2020)	Phase II	NCT01610206	Persistent or recurrent epithelial ovarian, fallopian tube or primary peritoneal carcinoma	75/73	63	VEGFR inhibitor	Gem (1,000 mg/m^2^, weekly on days 1 and 8, every 21 days) + Paz (800 mg, orally, daily)	Gem (1,000 mg/m^2^, weekly on days 1 and 8, every 21 days)	13	1
Aghajanian et al. (2015)	Phase III	OCEANS	Platinum-sensitive, recurrent epithelial ovarian, fallopian tube, or primary peritoneal carcinoma; ECOG PS of 0–1	242/242	60/61	VEGF inhibitor	Cycles 1-6: Gem (1,000 mg/m^2^, days 1 and 8) + Car (AUC 4, day 1) + Bev (15 mg/kg on day 1, 6-10 cycles of 21 days); Cycles 10+: Bev (15 mg/kg)	Cycles 1-6: Gem (1,000 mg/m^2^, days 1 and 8) and Car (AUC 4, day 1) + PL (15 mg/kg on day 1,6-10 cycles of 21 days); Cycles 10+: PL (15 mg/kg)	9.6/8.4	2, 4, 5
Roque et al. (2022)	Phase II	NCT03093155	Platinum-resistant or refractory epithelial (non-mucinous) ovarian, fallopian tube, or primary peritoneal carcinoma; ECOG PS of 0–2	39/37	67/67	VEGF inhibitor	Ixa (20 mg/m^2^, i.v., days 1, 8, and 15 of a 28-day cycle) + Bev (10 mg/kg, i.v., days 1, 15 every 28 days)	Ixa (20 mg/m^2^, i.v., days 1, 8, and 15 of a 28-day cycle)	NA	1, 2, 3
Ledermann et al. (2016)	Phase III	ICON6	Platinum-sensitive, relapsed, epithelial ovarian cancer, primary peritoneal carcinomatosis or fallopian tube cancer after first-line platinum-based chemotherapy; ECOG PS of 0–1	164/118	62/62	VEGFR inhibitor	Platinum-based chemotherapy + Ced (20 mg, qd) then maintenance Ced (20 mg, qd) alone	Platinum-based chemotherapy + PL (20 mg, qd) then maintenance PL (20 mg, qd) alone	19.5	1
Shoji et al. (2022)	Phase II	JGOG3023	Platinum-resistant, epithelial ovarian, fallopian tube, or primary peritoneal carcinoma; ECOG PS of 0–2	52/51	60.3 (mean age)/60.7 (mean age)	VEGF inhibitor	Chemotherapy (PLD/Top/Pac/Gem) + Bev (i.v., 15 mg/kg)	Chemotherapy (PLD/Top/Pac/Gem)	NA	1, 2, 3, 4, 5
Pignata et al. (2015)	Phase II	MITO 11	Platinum-resistant or refractory ovarian cancer; ECOG PS of 0–1	37/36	56/58	VEGFR inhibitor	Pac (80 mg/m^2^ on days 1, 8 and 15 in every 28 days) + Paz (800 mg daily)	Pac (80 mg/m^2^ on days 1, 8 and 15 every 28 days)	16.3/16.1	1, 2, 3, 5
Chekerov et al. (2018)	Phase II	TRIAS	Platinum-resistant ovarian, peritoneal, or fallopian tube cancers; ECOG PS of 0–2	83/89	59/58	VEGFR inhibitor	Cycles 1-6: Top (1–25 mg/m^2^ on days 1–5) + Sor (400 mg oral bid on days 6–15, every 21 days); Cycles 6+: Daily maintenance Sor for up to 1 year	Cycles 1-6: Top (1–25 mg/m^2^ on days 1–5) + PL (bid on days 6–15, every 21 days); Cycles 6+: Daily maintenance PL for up to 1 year	11.3/8.7	1, 2, 3, 4, 5
Wang et al. (2022)	Phase II	APPROVE	Platinum-resistant, recurrent epithelial ovarian cancer, primary peritoneal cancer, or fallopian tube cancer; ECOG PS of 0–1	78/74	54/56	VEGFR inhibitor	PLD (i.v., 40 mg/m^2^, q4w, up to 6 cycles) + Apa (orally, 250 mg, qd, up to 6 cycles)	PLD (i.v., 40 mg/m^2^, q4w, up to 6 cycles)	8.7	1, 2, 3, 4, 5
Pujade-Lauraine et al. (2014)	Phase III	AURELIA	Platinum-resistant, recurrent epithelial ovarian, fallopian tube or primary peritoneal cancer; ECOG PS of 0–2	179/182	62/61	VEGF inhibitor	Chemotherapy (PLD/Pac/Top) + Bev (15 mg/kg, q3w or 10 mg/kg, q2w)	Chemotherapy (PLD/Pac/Top)	13.0/13.9	1, 2, 3
Ledermann et al. (2021)	Phase III	ICON6	Platinum-sensitive, relapsed, epithelial ovarian cancer, primary peritoneal carcinomatosis or fallopian tube cancer after first-line platinum-based chemotherapy; ECOG PS of 0–1	164/118	62/62	VEGFR inhibitor	Platinum-based chemotherapy + Ced (20 mg, qd) then maintenance Ced (20 mg, qd) alone	Platinum-based chemotherapy + PL (20 mg, qd) then maintenance PL (20 mg, qd) alone	25.6	2
Aghajanian et al. (2012)	Phase III	OCEANS	Platinum-sensitive, recurrent epithelial ovarian, fallopian tube, or primary peritoneal carcinoma; ECOG PS of 0–1	242/242	60.5/61.6	VEGF inhibitor	Cycles 1-10: Gem (1,000 mg/m^2^ on days 1 and 8) + Car (AUC 4 on day 1) + Bev (15 mg/kg on day 1), q3w	Cycles 1-10: Gem (1,000 mg/m^2^, days 1 and 8) + Car (AUC 4, day 1) + PL (15 mg/kg, day 1), q3w	24	1, 3
Hall et al. (2020)	Phase II	NCT01610869	Platinum resistant or intolerant ovarian, fallopian tube or primary peritoneal carcinoma	59/55	62.4/65.7	VEGFR inhibitor	Cyc (orally, 100 mg, qd, in cycles of 6 weeks) + Nin (200 mg, bid)	Cyc (orally, 100 mg, qd, in cycles of 6 weeks)	19.2	1, 2, 3, 4, 5

E, experimental arm; C, control arm; GOG, the Gynecologic Oncology Group; PS, performance status; VEGF, vascular endothelial growth factor; Pac, paclitaxel; Car, carboplatin; AUC, area under curve; Bev, bevacizumab; q3w, every 3 weeks; FIGO, international federation of gynecology and obstetrics; ECOG, eastern cooperative oncology group; VEGFR, vascular endothelial growth factor receptor; Paz, pazopanib; PL, placebo; Gem, gemcitabine; qd, once daily; PLD, pegylated liposomal doxorubicin; i.v., intravenously; Apa, Apatinib; NA, not available; Top, topotecan; Sor, sorafenib; bid, twice daily; Nin, nintedanib; Ixa, ixabepilone; Cyc, cyclophosphamide; 1, progression-free survival; 2, overall survival; 3, objective response rate; 4, any grade adverse events; 5, grade ≥3 adverse events.

### 3.3 Overall analysis of platinum-sensitive ovarian cancer

**FIGURE 2:**
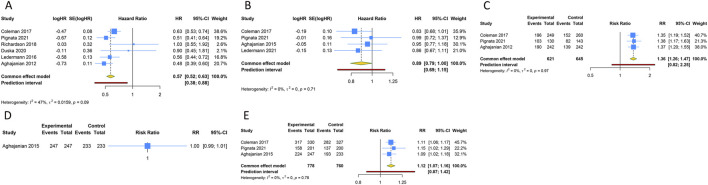
Forest plot of the efficacy and safety outcomes after anti-angiogenic drugs combined with chemotherapy for platinum-sensitive ovarian cancer. **(A)** Progression-free survival; **(B)** Overall survival; **(C)** Objective response rate; **(D)** Any grade adverse events; **(E)** Grade ≥3 adverse events.

5 RCTs were undertaken to gauge the PFS advantage of uniting anti-angiogenic medications with CT in the treatment of PSOC patients. Given the absence of significant heterogeneity across the trials, analysis was conducted using a fixed-effects model (I^2^ = 47.1%, Tau^2^ = 0.0159). The amalgamated estimate demonstrated a significant PFS benefit when anti-angiogenic drugs were used in conjunction with CT, compared to CT administered alone or combined with a placebo (HR [95% CI] = 0.573 [0.518–0.633], 95% PI: 0.383–0.876). However, combined findings from a fixed-effects model (I^2^ = 0%, Tau^2^ = 0), based on 4 RCTs, indicated that the addition of anti-angiogenic drugs to CT did not significantly amplify OS (HR [95% CI] = 0.891 [0.794–1.001], 95% PI: 0.691–1.149). In addition, 3 studies presented the ORR outcome in PSOC patients, demonstrating that the ORR for the combination of anti-angiogenic agents and CT was significantly superior to that of CT alone (or with a placebo) (RR [95% CI] = 1.362 [1.260–1.472], 95% PI: 0.824–2.251; I^2^ = 0%, Tau^2^ = 0). One study provided information on AEs of any grade associated with PSOC patients (Aghajanian et al., 2015), indicating no significant disparity in the risk of any grade AEs between the group receiving anti-angiogenic agents with CT and the control group (RR [95% CI] = 1.000 [0.992–1.008]). Regarding AEs of grade 3 or above, the consolidated results from 3 trials indicated that the incidence of grade ≥3 AEs was significantly elevated when anti-angiogenic agents were used in combination with CT, as compared to CT administered alone or with placebo (RR [95% CI] = 1.115 [1.070–1.162], 95% PI: 0.870–1.422; I^2^ = 0%, Tau^2^ = 0) (Table 2; Figure 2).

**TABLE 2:** Pooled effect of the efficacy and safety of anti-angiogenic drugs combined with chemotherapy in the treatment of platinum-sensitive/resistant ovarian cancer.

Outcomes	Number of studies	Meta-analysis				Heterogeneity	
		HR/RR	95% CI	*p*-value	95% PI	I^2^, tau^2^	*p*-value
Platinum-sensitive ovarian cancer							
PFS	6	0.573	0.518–0.633	<0.001	0.383-0.876	47.1%, 0.0159	0.092
OS	4	0.891	0.794–1.001	0.051	0.691-1.149	0%, 0	0.705
ORR	3	1.362	1.260–1.472	<0.001	0.824-2.251	0%, 0	0.967
Any grade AEs	1	1.000	0.992–1.008	0.977			
Grade ≥3 AEs	3	1.115	1.070–1.162	<0.001	0.870-1.422	0%, 0	0.784
Platinum-resistant ovarian cancer							
PFS	9	0.542	0.475–0.619	<0.001	0.322-0.930	47.6%, 0.0402	0.054
OS	7	0.752	0.646–0.875	<0.001	0.554-0.997	11.4%, 0.0059	0.343
ORR	7	2.141	1.702–2.694	<0.001	0.839-5.307	49.1%, 0.0985	0.067
Any grade AEs	4	1.020	0.972–1.070	0.432	0.836-1.244	69.6%, 0.0015	0.020
Grade ≥3 AEs	5	1.487	1.216–1.819	<0.001	0.755-2.828	32.1%, 0.0263	0.207

PFS, progression-free survival; OS, overall survival; ORR, objective response rate; AEs, adverse events.

### 3.4 Subgroup analysis of platinum-sensitive ovarian cancer

Subgroup analysis was performed according to the types of anti-angiogenic drugs. The analysis suggested that VEGF inhibitors combined with CT significantly improved PFS (HR [95% CI] = 0.546 [0.461–0.647], 95% PI: 0.093–3.190; I^2^ = 52.3%, Tau^2^ = 0.0118), but did not result in an OS improvement (HR [95% CI] = 0.900 [0.790–1.025], 95% PI: 0.387–2.094; I^2^ = 0%, Tau^2^ = 0) compared with CT alone (or plus placebo) in PSOC patients. Furthermore, it was observed that the combined treatment of VEGF inhibitors and CT improved ORR (RR [95% CI] = 1.362 [1.260–1.472], 95% PI: 0.824–2.251; I^2^ = 0%, Tau^2^ = 0) and escalated the occurrence of grade ≥3 AEs (RR [95% CI] = 1.115 [1.070–1.162], 95% PI: 0.870–1.422; I^2^ = 0%, Tau^2^ = 0). Turning to VEGFR inhibitors, the subgroup analyses failed to identify any PFS (HR [95% CI] = 0.733 [0.480–1.121], 95% PI: 0.008-63.683; I^2^ = 53.1%, Tau^2^ = 0.0766) or OS (HR [95% CI] = 0.860 [0.668–1.107]) benefits when these inhibitors were combined with CT. There were no available data on the effects of VEGFR inhibitors combined with CT on ORR, AEs of any grade, and grade ≥3 AEs in PSOC patients.

Further subgroup analysis indicated that the combination of pazopanib and CT did not significantly enhance PFS (HR [95% CI] = 0.971 [0.609–1.546]; I^2^ = 0%, Tau^2^ = 0). Only a single study presented the outcomes of combining cediranib and CT in terms of PFS and OS. The findings revealed that the combination of CT and cediranib significantly improved PFS (HR [95% CI] = 0.560 [0.438-0.716]), but had no significant impact on OS (HR [95% CI] = 0.860 [0.668–1.107]). Given that bevacizumab was the sole VEGF inhibitor used for PSOC patients in the included studies, the results of the analysis of bevacizumab combined with CT were in line with those of VEGF inhibitors (Table 3; Supplementary Figures S1–S5).

**TABLE 3:** Subgroup analysis of the efficacy and safety of anti-angiogenic drugs combined with chemotherapy in the treatment of platinum-sensitive/resistant ovarian cancer.

Subtypes	Number of studies	Meta-analysis				Heterogeneity	
		HR/RR	95% CI	*p*-value	95% PI	I^2^, tau^2^	*p*-value
Platinum-sensitive ovarian cancer							
PFS							
VEGF inhibitors + CT vs. CT (alone or + PL)	3	0.546	0.461–0.647	<0.001	0.093–3.190	52.3%, 0.0118	0.123
Bevacizumab + CT vs. CT (alone or + PL)	3	0.546	0.461–0.647	<0.001	0.093–3.190	52.3%, 0.0118	0.123
VEGFR inhibitors + CT vs. CT (alone or + PL)	3	0.733	0.480–1.121	0.152	0.008–63.683	53.1%, 0.0766	0.118
Pazopanib + CT vs. CT (alone or + PL)	2	0.971	0.609–1.546	0.900	-	0%, 0	0.778
Cediranib + CT vs. CT + PL	1	0.560	0.438–0.716	<0.001			
OS							
VEGF inhibitors + CT vs. CT (alone or + PL)	3	0.900	0.790–1.025	0.112	0.387–2.094	0%, 0	0.521
Bevacizumab + CT vs. CT (alone or + PL)	3	0.900	0.790–1.025	0.112	0.387–2.094	0%, 0	0.521
VEGFR inhibitors + CT vs. CT + PL	1	0.860	0.668–1.107	0.242			
Cediranib + CT vs. CT + PL	1	0.860	0.668–1.107	0.242			
ORR							
VEGF inhibitors + CT vs. CT (alone or + PL)	3	1.362	1.260–1.472	<0.001	0.824-2.251	0%, 0	0.967
Bevacizumab + CT vs. CT (alone or + PL)	3	1.362	1.260–1.472	<0.001	0.824–2.251	0%, 0	0.967
Any grade AEs							
VEGF inhibitors + CT vs. CT + PL	1	1.000	0.992–1.008	0.977			
Bevacizumab + CT vs. CT + PL	1	1.000	0.992–1.008	0.977			
Grade ≥ 3 AEs							
VEGF inhibitors + CT vs. CT (alone or + PL)	3	1.115	1.070–1.162	<0.001	0.870–1.422	0%, 0	0.784
Bevacizumab + CT vs. CT (alone or + PL)	3	1.115	1.070-1.162	<0.001	0.870–1.422	0%, 0	0.784
Platinum-resistant ovarian cancer							
PFS							
VEGF inhibitors + CT vs. CT	3	0.464	0.382–0.564	<0.001	0.132–1.637	0%, 0	0.369
Bevacizumab + CT vs. CT	3	0.464	0.382–0.564	<0.001	0.132–1.637	0%, 0	0.369
VEGFR inhibitors + CT vs. CT (alone or + PL)	6	0.620	0.518–0.742	<0.001	0.319–1.172	42.6%, 0.0391	0.121
Pazopanib + CT vs. CT (alone or + PL)	3	0.572	0.416–0.785	0.001	0.038–8.684	15.5%, 0.0145	0.306
Sorafenib + CT vs. CT + PL	1	0.600	0.432–0.834	0.002			
Apatinib + CT vs. CT	1	0.440	0.276–0.701	0.001			
Nintedanib + CT vs. CT	1	0.910	0.624–1.328	0.625			
OS							
VEGF inhibitors + CT vs. CT	3	0.761	0.619–0.935	0.010	0.044–11.812	34.7%, 0.0257	0.216
Bevacizumab + CT vs. CT	3	0.761	0.619–0.935	0.010	0.044–11.812	34.7%, 0.0257	0.216
VEGFR inhibitors + CT vs. CT (alone or + PL)	4	0.742	0.594–0.927	0.009	0.357–1.532	18.5%, 0.0123	0.298
Pazopanib + CT vs. CT	1	0.600	0.319–1.128	0.113			
Sorafenib + CT vs. CT + PL	1	0.650	0.452–0.934	0.020			
Apatinib + CT vs. CT	1	0.660	0.400-1.090	0.104			
Nintedanib + CT vs. CT	1	1.030	0.687–1.544	0.886			
ORR							
VEGF inhibitors + CT vs. CT	3	2.458	1.700–3.553	<0.001	0.220–26.606	0%, 0	0.538
Bevacizumab + CT vs. CT	3	2.458	1.700–3.553	<0.001	0.220–26.606	0%, 0	0.538
VEGFR inhibitors + CT vs. CT (alone or + PL)	4	1.967	1.165–3.319	0.011	0.220–17.583	66.6%, 0.1880	0.029
Pazopanib + CT vs. CT	1	2.222	1.176–4.201	0.014			
Sorafenib + CT vs. CT + PL	1	1.916	1.081–3.395	0.026			
Apatinib + CT vs. CT	1	3.939	1.855–8.362	<0.001			
Nintedanib + CT vs. CT	1	1.079	0.660–1.765	0.761			
Any grade AEs							
VEGF inhibitors + CT vs. CT	1	0.981	0.929–1.035	0.483			
Bevacizumab + CT vs. CT	1	0.981	0.929–1.035	0.483			
VEGFR inhibitors + CT vs. CT (alone or + PL)	3	1.050	1.003–1.100	0.037	0.629–1.704	47.2%, 0.0009	0.151
Sorafenib + CT vs. CT + PL	1	1.034	0.973-1.099	0.282			
Apatinib + CT vs. CT	1	1.101	0.980–1.236	0.105			
Nintedanib + CT vs. CT	1	1.019	0.970–1.070	0.455			
Grade ≥ 3 AEs							
VEGF inhibitors + CT vs. CT	1	1.279	0.876–1.866	0.202			
Bevacizumab + CT vs. CT	1	1.279	0.876–1.866	0.202			
VEGFR inhibitors + CT vs. CT (alone or + PL)	4	1.562	1.232–1.980	<0.001	0.431–5.698	46.4%, 0.0581	0.133
Pazopanib + CT vs. CT	1	1.946	1.112–3.406	0.020			
Sorafenib + CT vs. CT + PL	1	1.336	0.613–2.911	0.466			
Apatinib + CT vs. CT	1	2.224	1.299–3.808	0.004			
Nintedanib + CT vs. CT	1	1.181	0.869–1.605	0.289			

PFS, progression-free survival; VEGF, vascular endothelial growth factor; CT, chemotherapy; PL, placebo; VEGFR, vascular endothelial growth factor receptor; OS, overall survival; ORR, objective response rate; AEs, adverse events.

### 3.5 Overall analysis of platinum-resistant ovarian cancer

**FIGURE 3:**
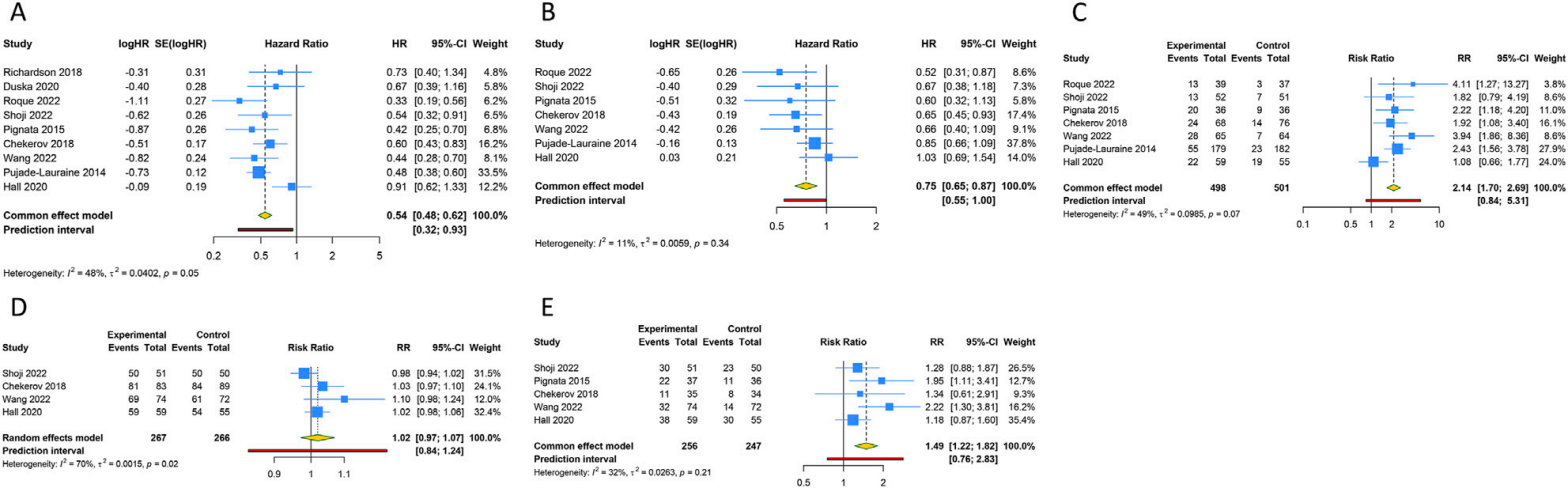
Forest plot of the efficacy and safety outcomes after anti-angiogenic drugs combined with chemotherapy for platinum-resistant ovarian cancer. **(A)** Progression-free survival; **(B)** Overall survival; **(C)** Objective response rate; **(D)** Any grade adverse events; **(E)** Grade ≥3 adverse events.

Nine RCTs assessed the PFS benefit of combining anti-angiogenic drugs with CT in patients with PROC. Due to the absence of significant heterogeneity among these studies concerning PFS, a fixed-effects model was applied to the pooled PFS analysis (I^2^ = 47.6%, Tau^2^ = 0.0402). The overall analysis demonstrated that the amalgamation of anti-angiogenic agents and CT resulted in a 45.8% reduction in the hazard of disease progression or death as compared to CT alone (or plus placebo) (HR [95% CI] = 0.542 [0.475–0.619], 95% PI: 0.322-0.930). Similarly, the consolidated data from a fixed-effects model (I^2^ = 11.4%, Tau^2^ = 0.0059), drawing from 7 RCTs, exhibited a noteworthy enhancement in OS when anti-angiogenic agents were co-administered with CT in a combined therapeutic approach *versus* the control (HR [95% CI] = 0.752 [0.646–0.875], 95% PI: 0.554–0.997). Additionally, 7 trials provided data on the ORR, with findings indicating a significantly higher ORR for the combination of anti-angiogenic agents and CT than for CT alone (or plus placebo) (RR [95% CI] = 2.141 [1.702–2.694], 95% PI: 0.839–5.307; I^2^ = 49.1%, Tau^2^ = 0.0985). In terms of AEs, the aggregated findings from 4 studies suggested no substantial disparity in the risk of AEs of any grade between the combination therapy cohort and the control group (RR [95% CI] = 1.020 [0.972–1.070], 95% PI: 0.836–1.244; I^2^ = 69.6%, Tau^2^ = 0.0015). Nevertheless, the incidence of grade ≥3 AEs was significantly elevated in the combination therapy cohort relative to the control group (RR [95% CI] = 1.487 [1.216–1.819], 95% PI: 0.755–2.828; I^2^ = 32.1%, Tau^2^ = 0.0263) (Table 2; Figure 3).

### 3.6 Subgroup analysis of platinum-resistant ovarian cancer

When classified by the types of anti-angiogenic agents, it was discerned that VEGF inhibitors combined with CT significantly improved PFS (HR [95% CI] = 0.464 [0.382-0.564], 95% PI: 0.132-1.637; I^2^ = 0%, Tau^2^ = 0) and OS (HR [95% CI] = 0.761 [0.619–0.935], 95% PI: 0.044–11.812; I^2^ = 34.7%, Tau^2^ = 0.0257) in PROC patients compared with CT alone, whilst also boosting the ORR (RR [95% CI] = 2.458 [1.700–3.553], 95% PI: 0.220-26.606; I^2^ = 0%, Tau^2^ = 0). Only a single study presented the outcomes of VEGF inhibitors in relation to AEs, suggesting no escalation in the risk for AEs of any grade (RR [95% CI] = 0.981 [0.929–1.035]) or grade ≥3 AEs (RR [95% CI] = 1.279 [0.876–1.866]) in PROC patients following treatment with a combination of VEGF inhibitors and CT. For VEGFR inhibitors, subgroup analysis revealed that their combination with CT significantly improved PFS (HR [95% CI] = 0.620 [0.518–0.742], 95% PI: 0.319-1.172; I^2^ = 42.6%, Tau^2^ = 0.0391) and OS (HR [95% CI] = 0.742 [0.594–0.927], 95% PI: 0.357–1.532; I^2^ = 18.5%, Tau^2^ = 0.0123), elevated ORR (RR [95% CI] = 1.967 [1.165–3.319], 95% PI: 0.220-17.583; I^2^ = 66.6%, Tau^2^ = 0.1880), but also increased the risks of any grade AEs (RR [95% CI] = 1.050 [1.003–1.100], 95% PI: 0.629-1.704; I^2^ = 47.2%, Tau^2^ = 0.0009) and grade ≥3 AEs (RR [95% CI] = 1.562 [1.232–1.980], 95% PI: 0.431-5.698; I^2^ = 46.4%, Tau^2^ = 0.0581).

Further subgroup analysis based on the types of VEGF or VEGFR inhibitors found that only one study reported the results of PFS, OS, ORR, and AEs after sorafenib, apatinib, or nintedanib combined with CT for treating PROC. The pooled results from 3 trials suggested that the addition of pazopanib to CT significantly improved PFS in comparison to CT alone or CT plus placebo (HR [95% CI] = 0.572 [0.416–0.785], 95% PI: 0.038–8.684; I^2^ = 15.5%, Tau^2^ = 0.0145). Only a single study showed the results of OS, ORR, and AEs following pazopanib treatment for PROC, respectively. In addition, as bevacizumab was the only VEGF inhibitor utilized in the treatment of PROC patients in the included studies, the corresponding results for bevacizumab treatment mirrored those of VEGF inhibitors (Table 3; Supplementary Figures S6–S10).

### 3.7 Sensitivity analysis and publication bias

We conducted sensitivity analyses and tests for publication bias on the amalgamated outcomes derived from more than 6 studies. The sensitivity analyses involved recalculating the combined HRs or RRs along with their 95% CIs, each time omitting a different individual study to verify the stability of the overall results. These analyses confirmed that the removal of any individual study did not markedly alter the overall conclusions, suggesting that the results derived from combining anti-angiogenic drugs with CT therapy are stable and reliable (Supplementary Figure S11). To assess the potential for publication bias, we employed Begg’s and Egger’s tests. The findings revealed no significant publication bias in the evaluated outcomes (all *p* > 0.05). The corresponding funnel plots were depicted in Supplementary Figure S12.

### 3.8 Trial sequential analysis results

**FIGURE 4:**
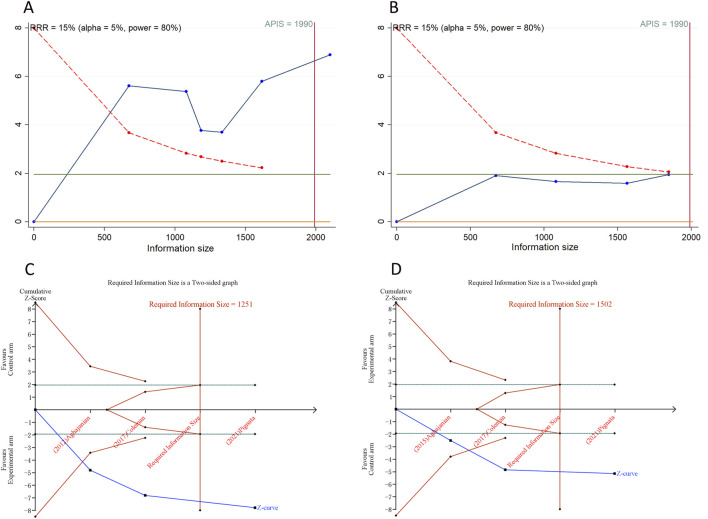
Trial sequential analysis of anti-angiogenic drugs combined with chemotherapy for platinum-sensitive ovarian cancer. **(A)** Progression-free survival; **(B)** Overall survival; **(C)** Objective response rate; **(D)** Grade ≥3 adverse events. Uppermost and lowermost red curves represent trial sequential monitoring boundary lines for benefit and harm, respectively. Inner red lines represent the futility boundary. Blue line represents evolution of cumulative Z-score. Horizontal green lines represent the conventional boundaries for statistical significance. Cumulative Z-curve crossing the trial sequential monitoring boundary or the RIS boundary provides firm evidence of effect.

**FIGURE 5:**
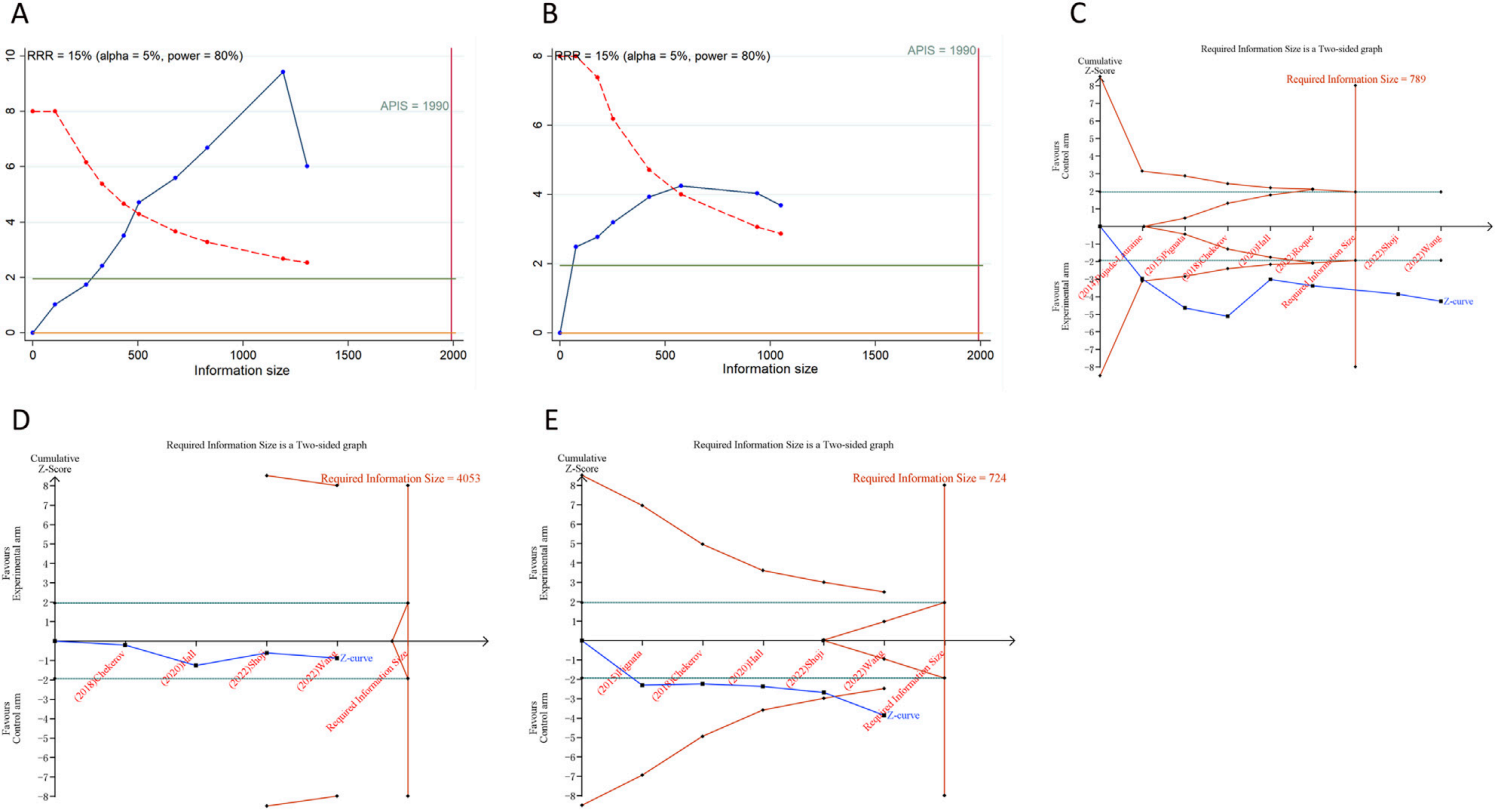
Trial sequential analysis of anti-angiogenic drugs combined with chemotherapy for platinum-resistant ovarian cancer. **(A)** Progression-free survival; **(B)** Overall survival; **(C)** Objective response rate; **(D)** Any grade adverse events; **(E)** Grade ≥3 adverse events. Uppermost and lowermost red curves represent trial sequential monitoring boundary lines for benefit and harm, respectively. Inner red lines represent the futility boundary. Blue line represents evolution of cumulative Z-score. Horizontal green lines represent the conventional boundaries for statistical significance. Cumulative Z-curve crossing the trial sequential monitoring boundary or the RIS boundary provides firm evidence of effect.

We calculated an APIS of 1,990 in the application of TSA for PFS and OS. For PSOC, it was observed that the cumulative Z-curves for PFS, ORR, and grade ≥ 3AEs surpassed both the trial sequential monitoring boundary and RIS threshold, suggesting the potential for robust conclusions. Conversely, the cumulative Z-curve for OS within the PSOC analysis did not intersect with either the trial sequential monitoring boundary or the RIS threshold, indicating that the results remain indeterminate and could potentially include false positives (Figure 4). In the context of PROC analysis, all cumulative Z-curves, with the exception of those for any AEs, crossed either the RIS threshold or the trial sequential monitoring boundary (Figure 5). This indicates that additional trials are unlikely to be needed to ascertain definitive results for PFS, OS, ORR, or grade ≥3 AEs. However, the results for any grade AEs in PROC patients would require additional studies and larger sample sizes to achieve validation.

## 4 Discussion

The management of recurrent OC is typically guided by numerous factors, amongst which the duration from the conclusion of platinum-based therapy to the identification of relapse stands as a critical factor (Petrelli et al., 2023). Traditionally, combination CT has been the regimen of choice as opposed to CT alone, given its higher ORR and enhanced PFS (Raja et al., 2013). This meta-analysis, encompassing 15 RCTs, revealed that compared with CT alone (or plus placebo), anti-angiogenic agents combined with CT significantly improved PFS and increased ORR, albeit accompanied by an escalated risk of grade ≥3 AEs among patients with PSOC. Likewise, this combination therapy also extended PFS and OS in patients with PROC, alongside an augmented ORR and a rise in the occurrence of grade ≥3 AEs.

Anti-angiogenic pharmaceuticals are categorized into three groups according to their mode of action: VEGF inhibitors such as bevacizumab, VEGFR inhibitors including pazopanib, cediranib, nintedanib, apatinib, and sorafenib, and angiopoietin inhibitors (Monk et al., 2016b). The VEGF and VEGFR pathways play a pivotal role in the regulation of angiogenesis, including in OC (Graybill et al., 2015). Our subgroup analysis disclosed that the combination of VEGF inhibitors and CT can markedly enhance PFS and escalate the ORR among PSOC patients. In patients with PROC, VEGF inhibitors combined with CT significantly improved both PFS and OS, and elevate ORR. Moreover, further analysis based on the types of VEGF inhibitors found that bevacizumab was the sole VEGF inhibitor employed for OC treatment in the included trials. Thus, the observed efficacy results of VEGF inhibitor combined with CT in treating PSOC or PROC in our study can be interpreted through the lens of bevacizumab’s role.

Bevacizumab, a humanized monoclonal antibody against VEGF, plays a pivotal role in angiogenesis inhibition. It holds the distinction of being the inaugural angiogenesis inhibitor utilized in oncological clinical practice, and the first to receive approval for treating OC patients (Marchetti et al., 2019). The U.S. Food and Drug Administration (FDA) has sanctioned bevacizumab as a first-line combination treatment with paclitaxel or carboplatin, and as a second-line strategy for PSOC or PROC (Burger et al., 2011; Perren et al., 2011; Rossi et al., 2017). The mechanism of bevacizumab involves the obstruction of circulating VEGF and VEGFR interaction, leading to the destruction of existing blood vessels, disruption of neovascularization, reduction of intratumoral pressure, and ultimately, the inhibition of angiogenesis (Mancuso et al., 2006). The integration of bevacizumab with CT has become a widely endorsed standard in OC clinical practice (An et al., 2021). The FDA, in 2016, approved the use of bevacizumab in conjunction with platinum-based CT for platinum-sensitive recurrent OC (Walsh, 2020), a decision based on the outcomes of the OCEANS and GOG-0213 trials. The OCEANS trial demonstrated that bevacizumab enhanced PFS and the ORR, albeit without a significant advantage to OS (Aghajanian et al., 2012). While the GOG-0213 trial indicated an improvement in OS with the combination of bevacizumab, paclitaxel, and carboplatin, followed by maintenance therapy with bevacizumab (Coleman et al., 2017). In cases where PROC advances after treatment with bevacizumab-inclusive regimens, the typical strategy is to implement single-agent non-platinum CT (Eskander et al., 2023). The JGOG3023 trial, a phase 2 study, investigated the effectiveness of single-agent non-platinum CT, with or without bevacizumab, in Japanese patients with PROC recurrence following a bevacizumab-inclusive CT regimen (Shoji et al., 2022). Despite patients treated with CT combined with bevacizumab exhibiting a numerically superior median OS compared to those receiving CT alone (15.3 vs. 11.3 months) and a higher ORR (25.0% vs. 13.7%), these results were not statistically significant, potentially attributed to the small sample size (Shoji et al., 2022). The findings of a recently published randomized research suggested that the addition of bevacizumab to CT post-platinum-sensitive relapse in patients previously treated with bevacizumab also significantly extended PFS (Pignata et al., 2018).

VEGFR inhibitors, representing another key class of anti-angiogenic agents, are widely employed in the clinical treatment of PSOC and PROC. VEGFR expression by the microvascular endothelial cells within malignant ovarian neoplasms and borderline growths indicates their viability as targets for novel treatment strategies (Arend et al., 2021; Spannuth et al., 2009). Our analysis encompassed several VEGFR inhibitors used in RCTs, including pazopanib, cediranib, sorafenib, apatinib, and nintedanib. Subgroup analyses revealed that combining VEGFR inhibitors with CT markedly enhanced PFS and OS, and elevated the ORR in PROC patients relative to CT alone or combined with placebo. However, this combination therapy did not extend to PSOC patients, where significant improvements in PFS and OS were not observed. Further detailed analysis based on the specific VEGFR inhibitors did not produce satisfactory results. Specifically, our study indicated that cediranib, when used with CT, significantly boosted PFS in PSOC patients. Furthermore, combinations of sorafenib or apatinib with CT not only improved PFS but also increased ORR in PROC patients. Nonetheless, the reliability of these meta-analysis findings, based solely on one study, is questionable and unconvincing. Despite the current evidence being insufficient to warrant the approval of these VEGFR inhibitors in combination with CT for clinical treatment of recurrent OC, numerous phase II studies have reported their therapeutic benefits. For instance, For instance, Hirte and colleagues assessed the efficacy of cediranib by measuring ORR in patients with persistent or recurrent OC post-initial CT, enrolling 74 patients split between PSOC and PROC cohorts, with the PSOC group showing a 23% partial response rate and a 51% stable disease rate, culminating in a 77% clinical benefit rate. While the platinum-resistant group did not exhibit confirmed responses, they did achieve a clinical benefit rate of 66% (Hirte et al., 2015). Further research included a randomized, placebo-controlled trial that evaluated nintedanib as a maintenance treatment after CT in patients with resistant or partially platinum-sensitive relapsed OC, which demonstrated an extension of PFS at 36 weeks relative to the placebo group (HR = 0.68, *p* = 0.07) (Ledermann et al., 2011). Additionally, a randomized phase II trial reported significant prolongation of PFS with pazopanib combined with paclitaxel in PROC patients (HR = 0.42, *p* = 0.0002) (Pignata et al., 2015). Of note, our pooled results from 3 studies in our study showed that pazopanib plus CT significantly improved PFS in PROC patients compared with CT alone (or plus placebo). However, only a single study has explored the correlation between pazopanib plus CT and OS or ORR in PROC patients. Consequently, more RCTs are needed in the future to ascertain the efficacy of various VEGFR inhibitors in the treatment of recurrent OC.

Our investigation, along with prior studies, underscore the clinical efficacy benefits of combining anti-angiogenic drugs with CT for patients with PSOC or PROC. Nevertheless, the escalated risk of AEs associated with this combination treatment warrants attention. Our findings suggested that the combination therapy of bevacizumab and CT increased the risk of grade ≥3 AEs in patients with PSOC. Similarly, the combination of VEGFR inhibitors and CT elevated the occurrence of AEs of any grade and grade ≥3 AEs in PROC patients. As per prior research, the potential for complications presents a significant concern when contemplating the inclusion of bevacizumab in CT (Marchetti et al., 2019). Given the unique profile of VEGF inhibition, which affects both normal and tumorous tissues as well as their interface, bevacizumab is associated with an increased risk for drug-related AEs. These include proteinuria, hypertension, bleeding, gastrointestinal perforations, wound healing disruption, and arterial and venous thrombosis (Wu et al., 2017). Common AEs linked with VEGFR inhibitors, such as apatinib, when used in combination with CT, encompass neutropenia, hypertension, oral mucositis, nausea or vomiting, and hand-foot syndrome (Huang et al., 2020). Hence, vigilant monitoring and management of these AEs during anti-angiogenic therapy are crucial to mitigate associated risks.

Our meta-analysis is subject to several limitations. First, the limited quantity of included studies led to instability in our subgroup analysis results, particularly concerning specific types of VEGFR inhibitors like cediranib, pazopanib, and apatinib. These results warrant further validation following the publication of additional relevant trials. Second, it is important to note that while the majority of the studies incorporated into our analysis were published in high-impact journals, certain inherent factors such as pharmaceutical industry sponsorship and an open-label design could potentially introduce bias. This includes publication bias, which might influence the overall findings. Third, despite the involvement of independent assessors and the rigorous data extraction and quality assessment process using the modified Jadad scale, the possibility of subjective biases in the evaluation of study quality and data extraction cannot be entirely ruled out. Fourth, TSA results underscore the need for future meta-analysis with larger sample sizes and a greater number of RCTs to validate the findings pertaining to OS in PSOC patients and any grade AEs in PROC patients.

## 5 Conclusion

In conclusion, our meta-analysis of RCTs revealed that compared with CT alone (or plus placebo), the combination therapy of anti-angiogenic drugs and CT yields a significant improvement in PFS and ORR for patients with PSOC. This combination treatment also extended to patients with PROC, manifesting in improved PFS, OS, and ORR. Concurrently, there was a notable escalation in the incidence of grade ≥3 AEs among both PSOC and PROC patients receiving this combination therapy. It is therefore imperative to exercise rigorous monitoring and proactive management of AEs during the course of anti-angiogenic treatment to minimize potential risks.

## Data Availability

The original contributions presented in the study are included in the article/Supplementary Material, further inquiries can be directed to the corresponding author.
