# Supplementary material for: Efficacy and safety of anti-angiogenic drugs combined with chemotherapy in the treatment of platinum-sensitive/resistant ovarian cancer: a meta-analysis with trial sequential analysis of randomized controlled trials
Source: Front Pharmacol. 2024 Nov 21;15:1446403. doi: 10.3389/fphar.2024.1446403 (PMC11617189; doi:10.3389/fphar.2024.1446403)
Supplement: Supplementary file 1 [file Table1.DOCX]

| **TABLE S1** Quality analysis of the included RCTs by modified Jadad scale. | | | | | | |
| --- | --- | --- | --- | --- | --- | --- |
| Study | Randomization | Randomization concealment | Double blind | Withdrawals and dropouts | Score | Study quality |
| Coleman (2017) | 2 | 2 | 0 | 1 | 5 | High |
| Pignata (2021) | 2 | 2 | 0 | 1 | 5 | High |
| Richardson (2018) | 2 | 2 | 2 | 1 | 7 | High |
| Duska (2020) | 1 | 1 | 0 | 1 | 3 | Low |
| Aghajanian (2015) | 2 | 2 | 2 | 1 | 7 | High |
| Roque (2022) | 2 | 2 | 0 | 1 | 5 | High |
| Ledermann (2016) | 2 | 2 | 2 | 1 | 7 | High |
| Shoji (2022) | 2 | 2 | 0 | 1 | 5 | High |
| Pignata (2015) | 2 | 2 | 0 | 1 | 5 | High |
| Chekerov (2018) | 2 | 2 | 2 | 1 | 7 | High |
| Wang (2022) | 2 | 2 | 0 | 1 | 5 | High |
| Pujade-Lauraine (2014) | 2 | 2 | 0 | 1 | 5 | High |
| Ledermann (2021) | 2 | 2 | 2 | 1 | 7 | High |
| Aghajanian (2012) | 2 | 2 | 2 | 1 | 7 | High |
| Hall (2020) | 1 | 1 | 0 | 1 | 3 | Low |
