# Supplementary material for: Efficacy and safety of anti-angiogenic drugs combined with chemotherapy in the treatment of platinum-sensitive/resistant ovarian cancer: a meta-analysis with trial sequential analysis of randomized controlled trials
Source: Front Pharmacol. 2024 Nov 21;15:1446403. doi: 10.3389/fphar.2024.1446403 (PMC11617189; doi:10.3389/fphar.2024.1446403)

**FIGURE S1** Subgroup analysis of progression-free survival after anti-angiogenic drugs combined with chemotherapy (CT) for platinum-sensitive ovarian cancer. (A) Subgroup = VEGF inhibitors + CT vs. CT (alone or + placebo [PL]); (B) Subgroup = Bevacizumab + CT vs. CT (alone or + PL); (C) Subgroup = VEGFR inhibitors + CT vs. CT (alone or + PL); (D) Subgroup = Pazopanib + CT vs. CT (alone or + PL); (E) Subgroup = Cediranib + CT vs. CT + PL.


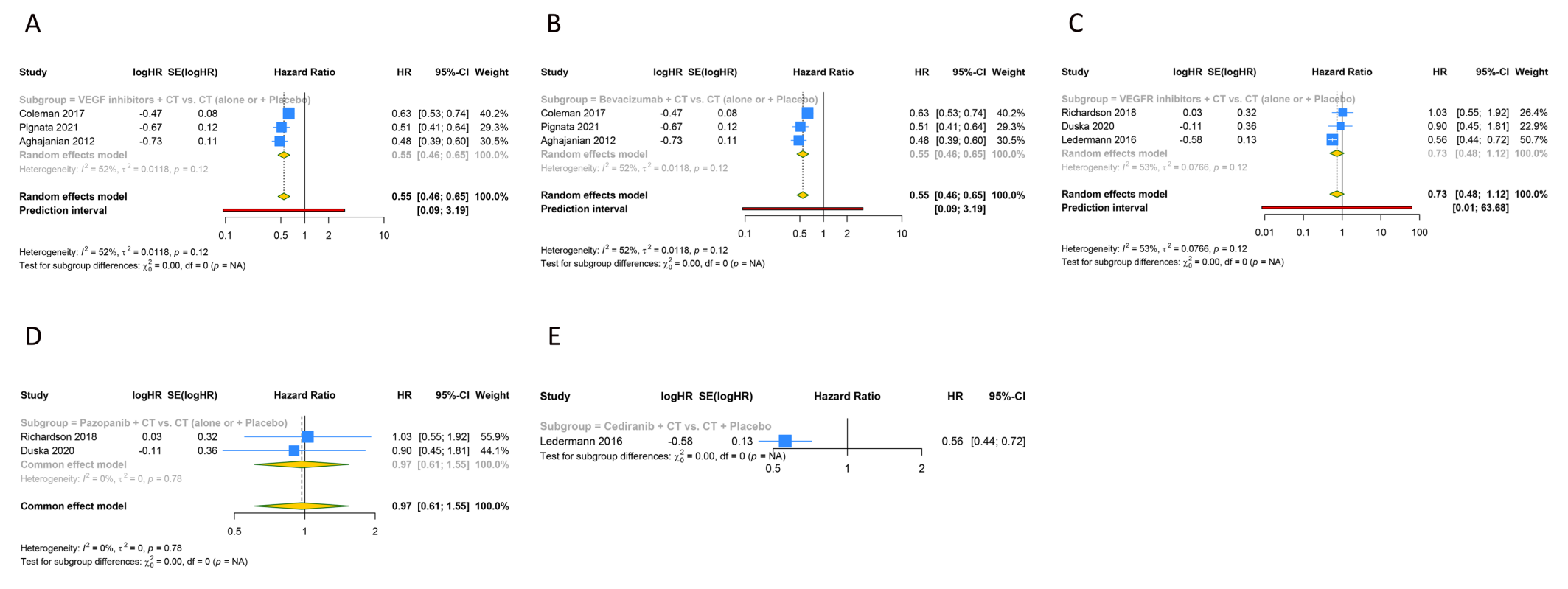


**FIGURE S2** Subgroup analysis of overall survival after anti-angiogenic drugs combined with chemotherapy (CT) for platinum-sensitive ovarian cancer. (A) Subgroup = VEGF inhibitors + CT vs. CT (alone or + placebo [PL]); (B) Subgroup = Bevacizumab + CT vs. CT (alone or + PL); (C) Subgroup = VEGFR inhibitors + CT vs. CT + PL; (D) Subgroup = Cediranib + CT vs. CT + PL.


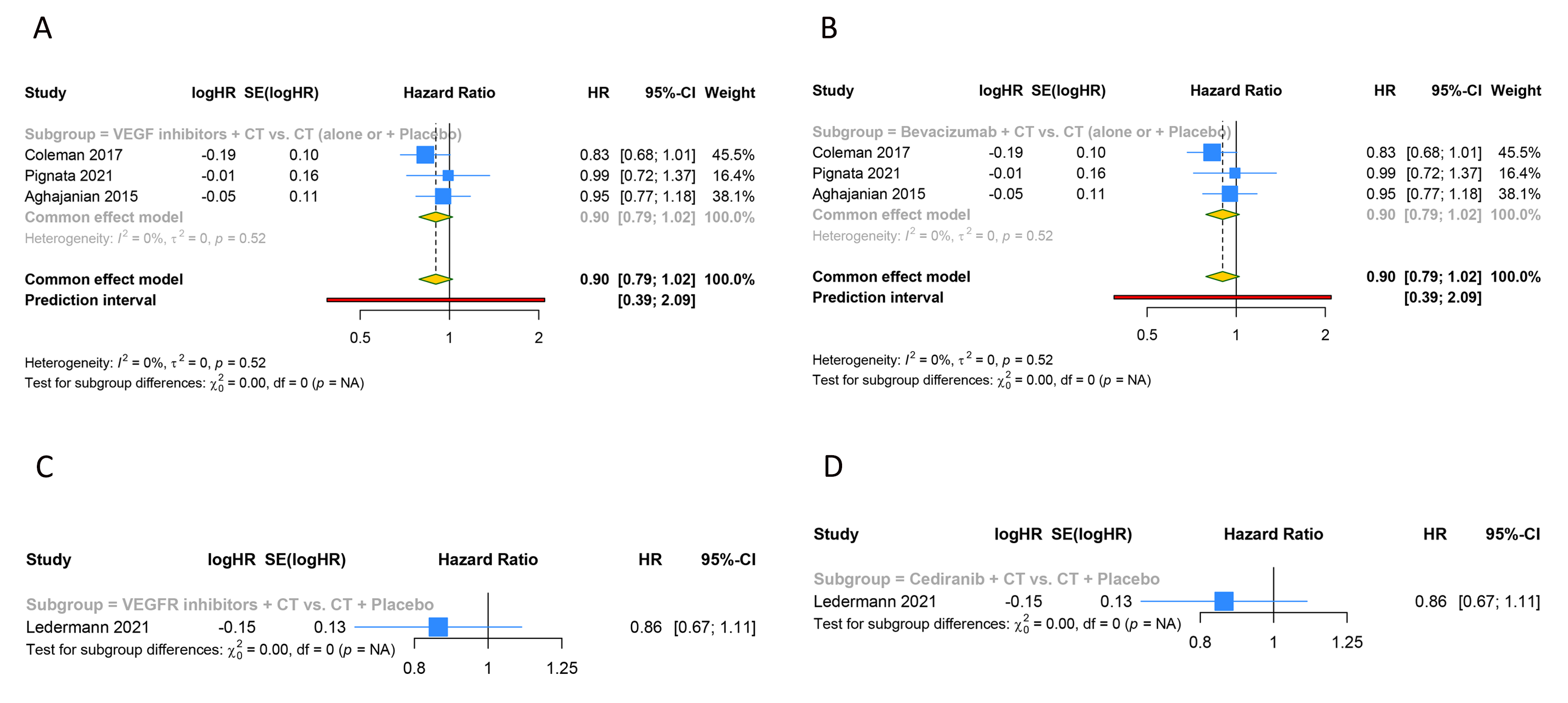


**FIGURE S3** Subgroup analysis of objective response rate after anti-angiogenic drugs combined with chemotherapy (CT) for platinum-sensitive ovarian cancer. (A) Subgroup = VEGF inhibitors + CT vs. CT (alone or + placebo [PL]); (B) Subgroup = Bevacizumab + CT vs. CT (alone or + PL).


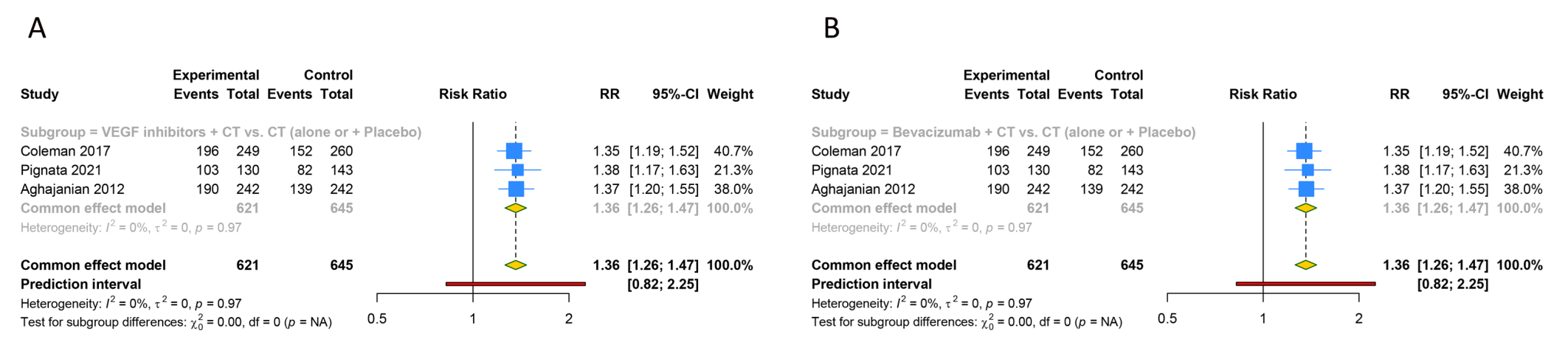


**FIGURE S4** Subgroup analysis of any grade adverse events after anti-angiogenic drugs combined with chemotherapy (CT) for platinum-sensitive ovarian cancer. (A) Subgroup = VEGF inhibitors + CT vs. CT + placebo (PL); (B) Subgroup = Bevacizumab + CT vs. CT + PL.


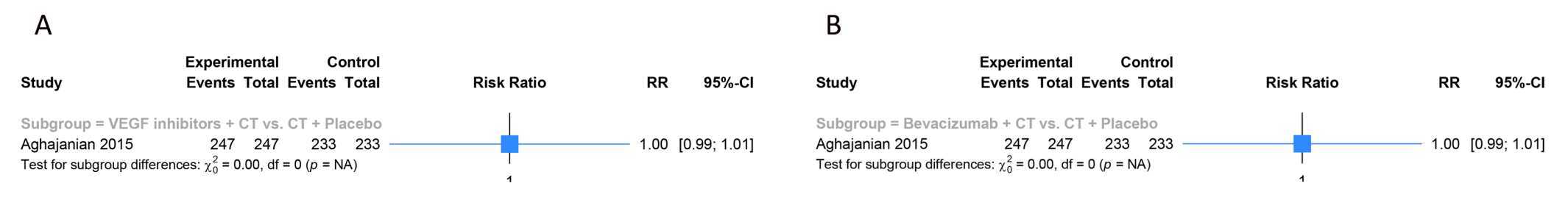


**FIGURE S5** Subgroup analysis of grade ≥ 3 adverse events after anti-angiogenic drugs combined with chemotherapy (CT) for platinum-sensitive ovarian cancer. (A) Subgroup = VEGF inhibitors + CT vs. CT (alone or + placebo [PL]); (B) Subgroup = Bevacizumab + CT vs. CT (alone or + PL).


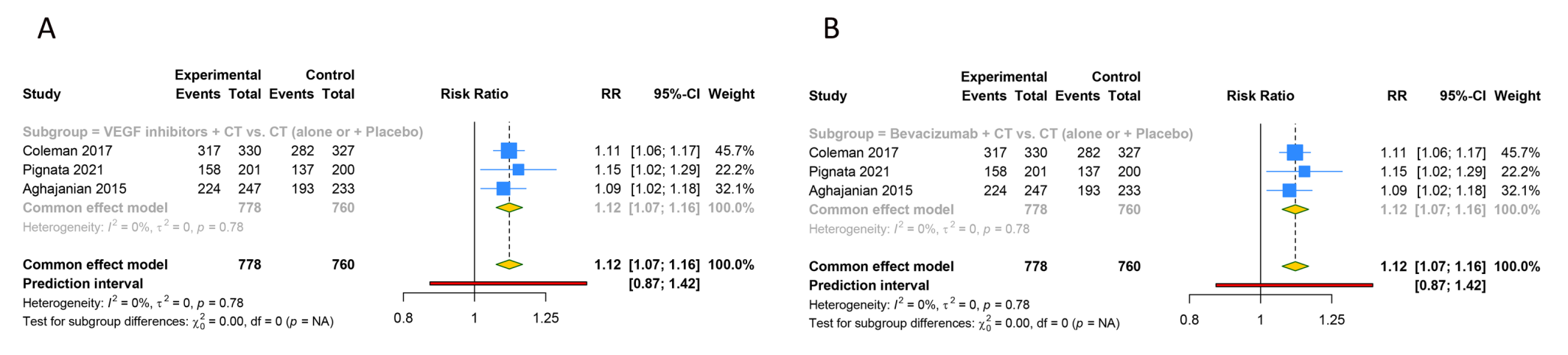


**FIGURE S6** Subgroup analysis of progression-free survival after anti-angiogenic drugs combined with chemotherapy (CT) for platinum-resistant ovarian cancer. (A) Subgroup = VEGF inhibitors + CT vs. CT; (B) Bevacizumab + CT vs. CT; (C) Subgroup = VEGFR inhibitors + CT vs. CT (alone or + placebo [PL]); (D) Subgroup = Pazopanib + CT vs. CT (alone or + PL); (E) Subgroup = Sorafenib + CT vs. CT + PL; (F) Subgroup = Apatinib + CT vs. CT; (G) Subgroup = Nintedanib + CT vs. CT.


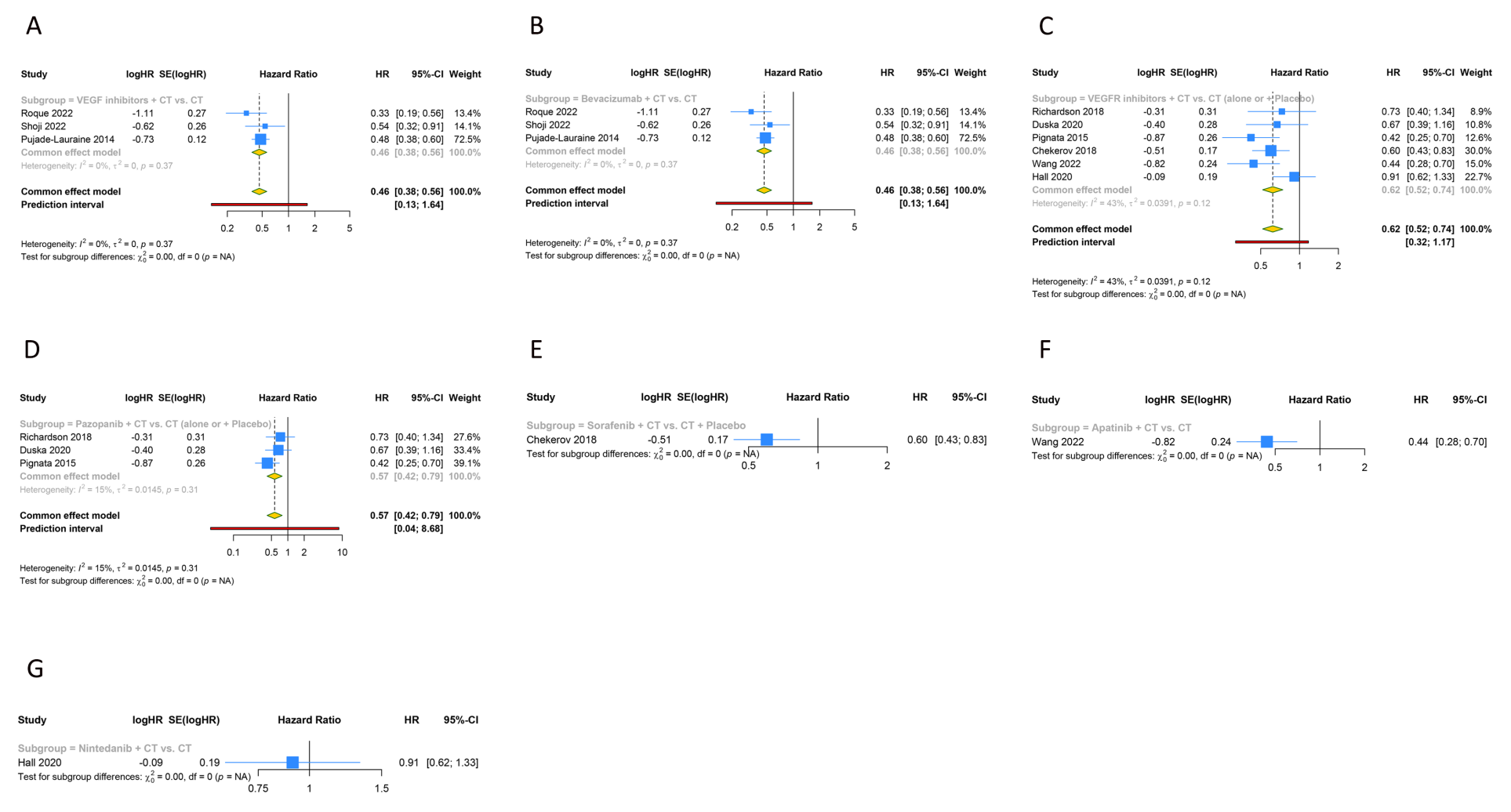


**FIGURE S7** Subgroup analysis of overall survival after anti-angiogenic drugs combined with chemotherapy (CT) for platinum-resistant ovarian cancer. (A) Subgroup = VEGF inhibitors + CT vs. CT; (B) Bevacizumab + CT vs. CT; (C) Subgroup = VEGFR inhibitors + CT vs. CT (alone or + placebo [PL]); (D) Subgroup = Pazopanib + CT vs. CT; (E) Subgroup = Sorafenib + CT vs. CT + PL; (F) Subgroup = Apatinib + CT vs. CT; (G) Subgroup = Nintedanib + CT vs. CT.


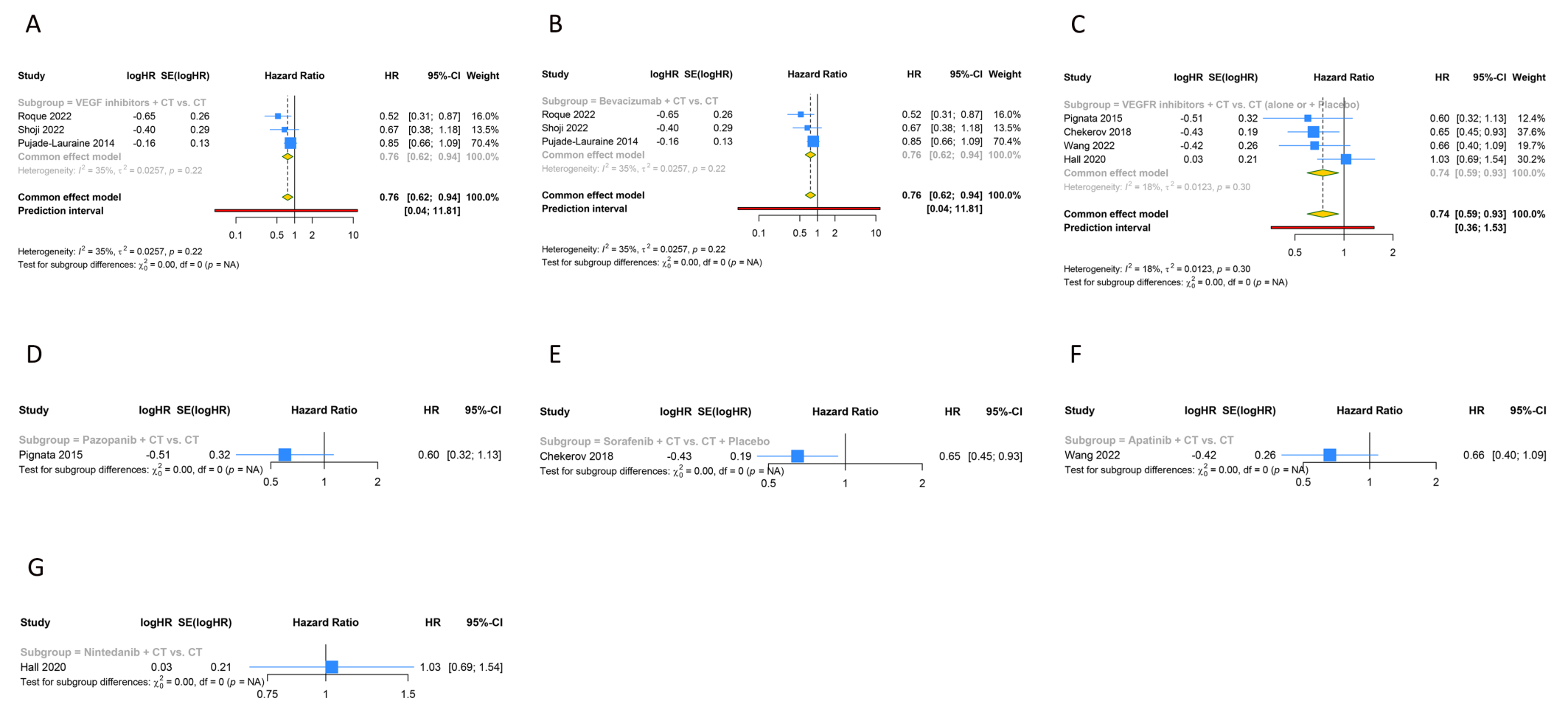


**FIGURE S8** Subgroup analysis of objective response rate after anti-angiogenic drugs combined with chemotherapy (CT) for platinum-resistant ovarian cancer. (A) Subgroup = VEGF inhibitors + CT vs. CT; (B) Bevacizumab + CT vs. CT; (C) Subgroup = VEGFR inhibitors + CT vs. CT (alone or + placebo [PL]); (D) Subgroup = Pazopanib + CT vs. CT; (E) Subgroup = Sorafenib + CT vs. CT + PL; (F) Subgroup = Apatinib + CT vs. CT; (G) Subgroup = Nintedanib + CT vs. CT.


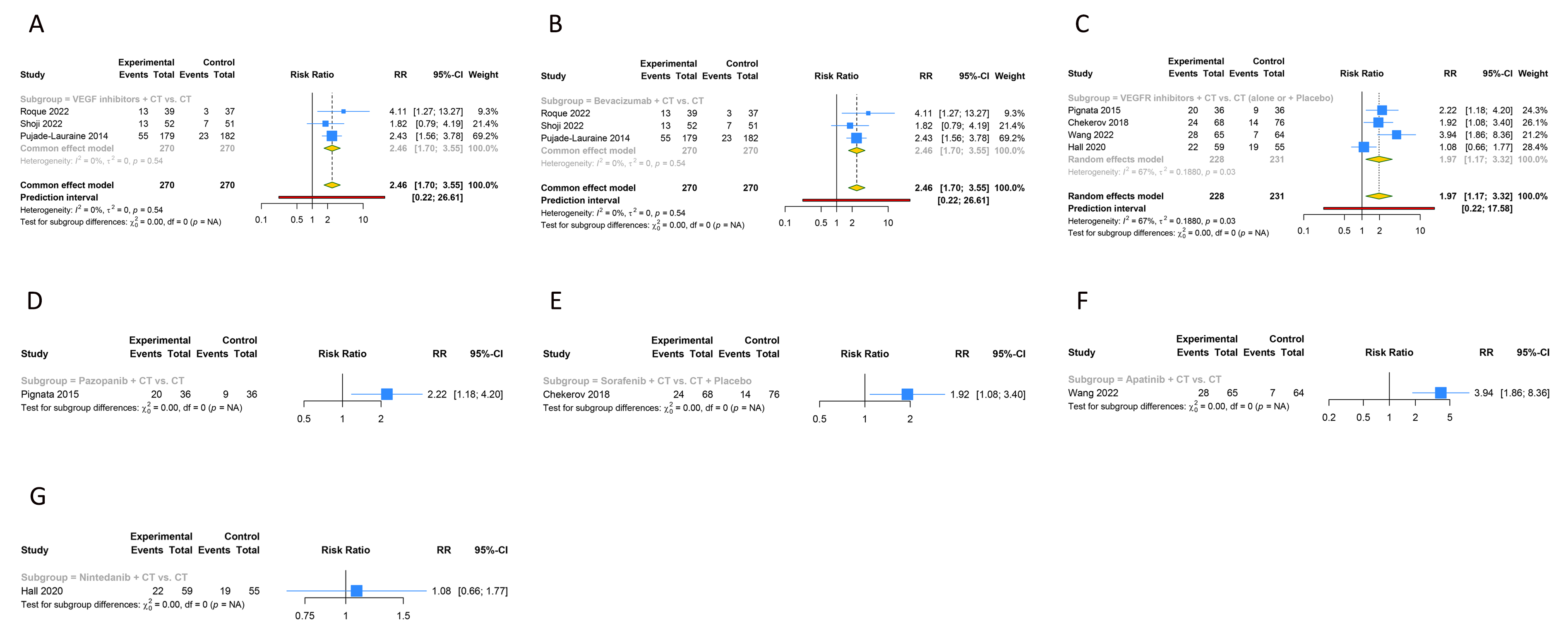


**FIGURE S9** Subgroup analysis of any grade adverse events after anti-angiogenic drugs combined with chemotherapy (CT) for platinum-resistant ovarian cancer. (A) Subgroup = VEGF inhibitors + CT vs. CT; (B) Bevacizumab + CT vs. CT; (C) Subgroup = VEGFR inhibitors + CT vs. CT (alone or + placebo [PL]); (D) Subgroup = Sorafenib + CT vs. CT + PL; (E) Subgroup = Apatinib + CT vs. CT; (F) Subgroup = Nintedanib + CT vs. CT.


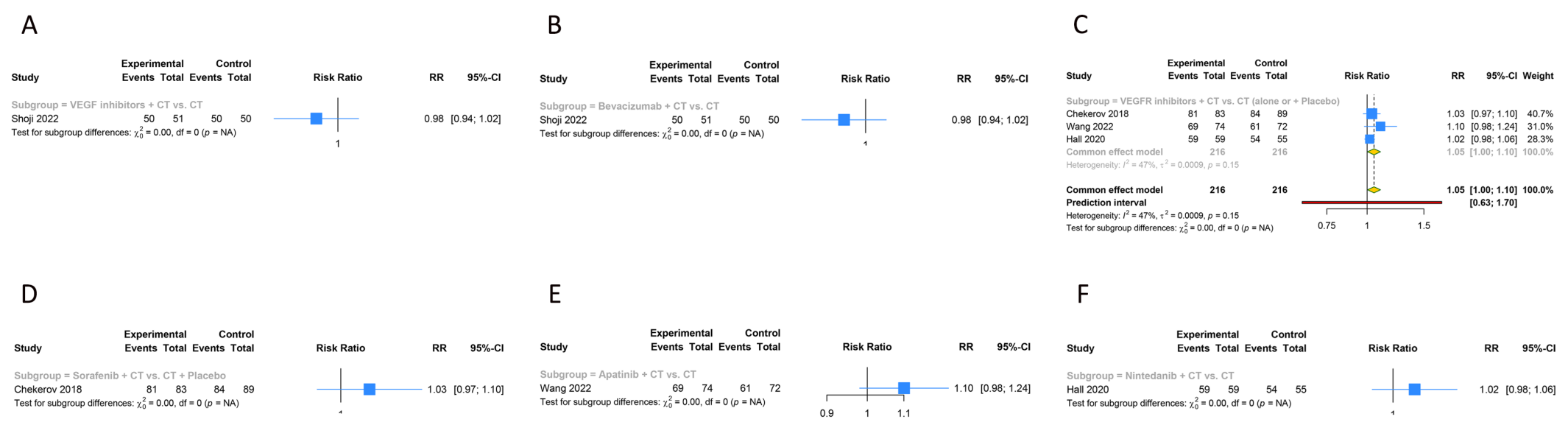


**FIGURE S10** Subgroup analysis of grade ≥ 3 adverse events after anti-angiogenic drugs combined with chemotherapy (CT) for platinum-resistant ovarian cancer. (A) Subgroup = VEGF inhibitors + CT vs. CT; (B) Bevacizumab + CT vs. CT; (C) Subgroup = VEGFR inhibitors + CT vs. CT (alone or + placebo [PL]); (D) Subgroup = Pazopanib + CT vs. CT; (E) Subgroup = Sorafenib + CT vs. CT + PL; (F) Subgroup = Apatinib + CT vs. CT; (G) Subgroup = Nintedanib + CT vs. CT.


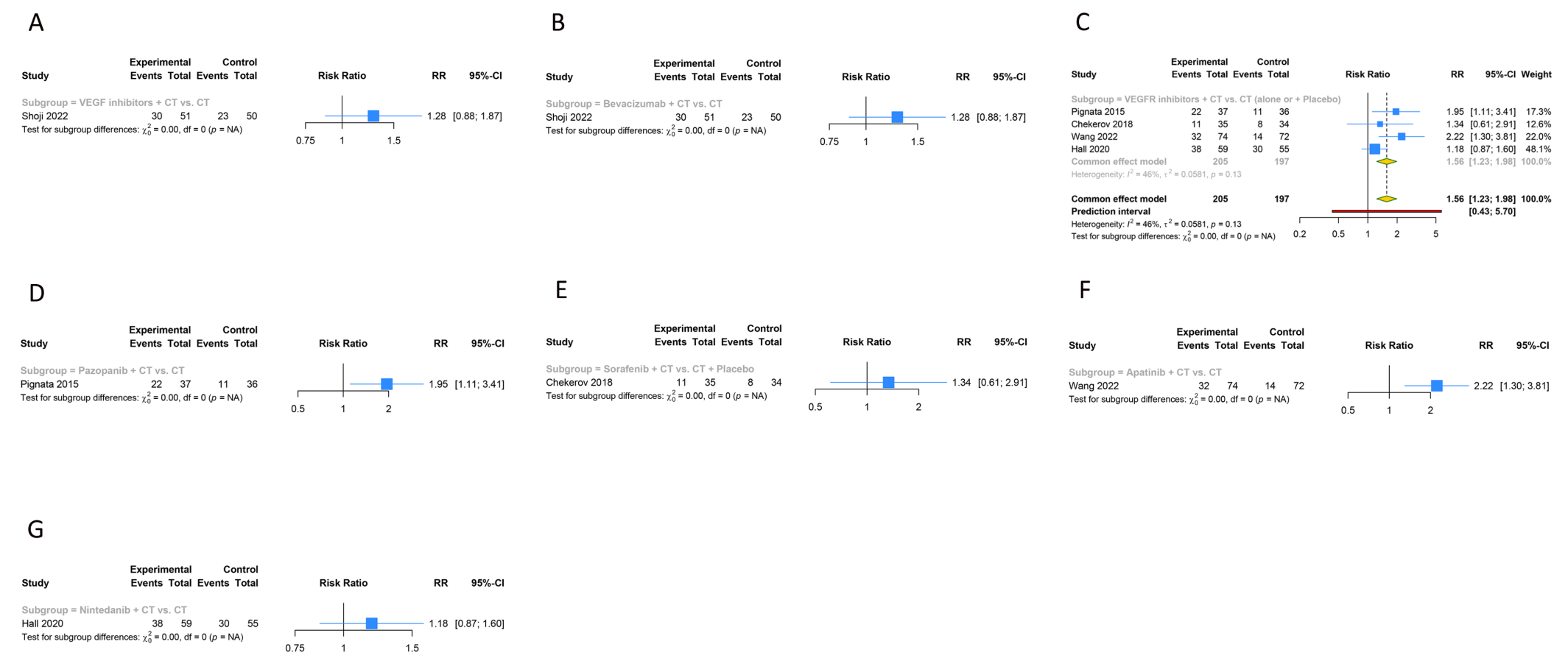


**FIGURE S11** Sensitivity analysis of anti-angiogenic drugs combined with chemotherapy for platinum-sensitive/resistant ovarian cancer. (A) Progression-free survival (PFS) for platinum-sensitive ovarian cancer; (B) PFS for platinum-resistant ovarian cancer (PROC); (C) Overall survival for PROC; (D) Objective response rate for PROC.


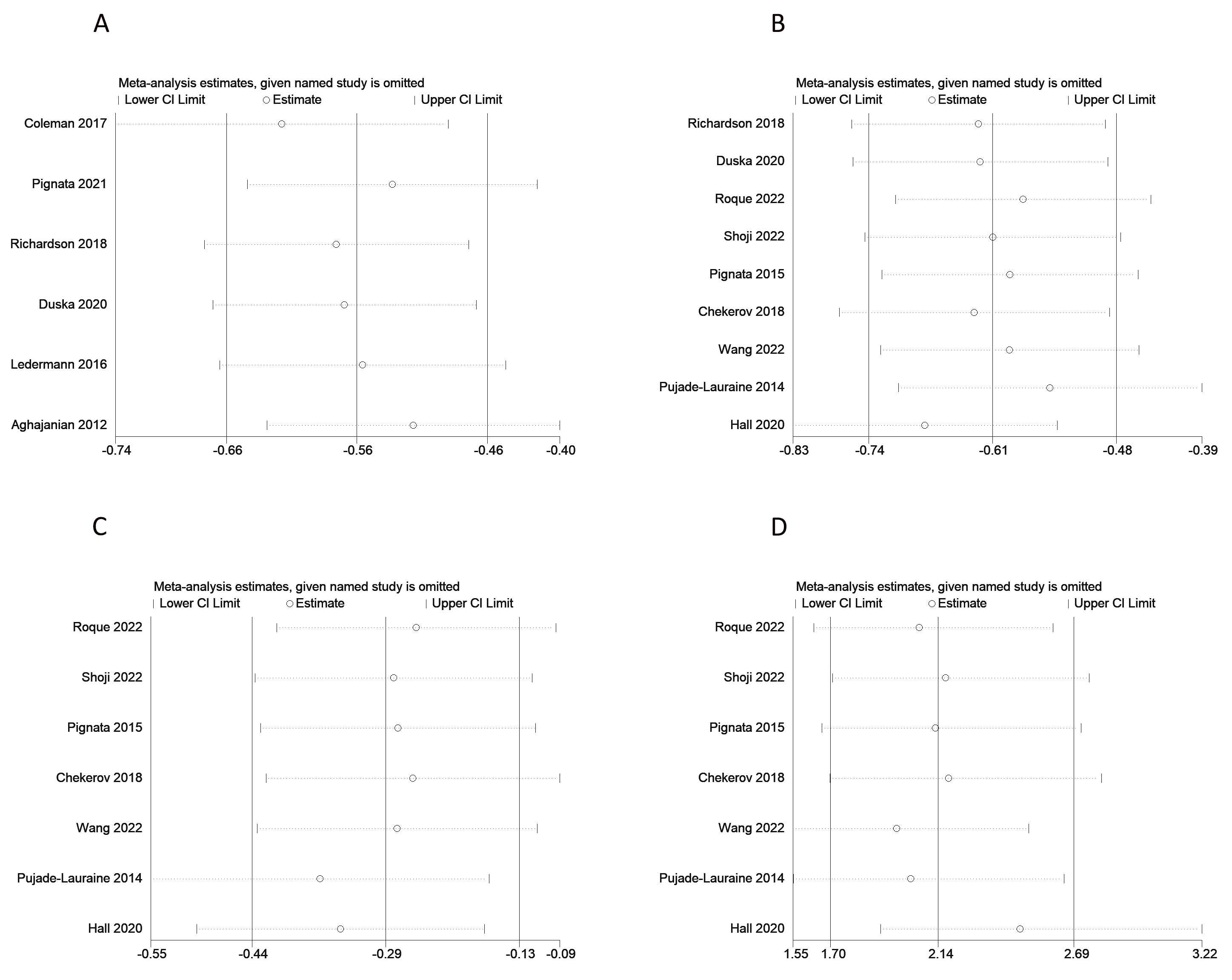


**FIGURE S12** Funnel plots of anti-angiogenic drugs combined with chemotherapy for platinum-sensitive/resistant ovarian cancer. (A) Progression-free survival (PFS) for platinum-sensitive ovarian cancer; (B) PFS for platinum-resistant ovarian cancer (PROC); (C) Overall survival for PROC; (D) Objective response rate for PROC.


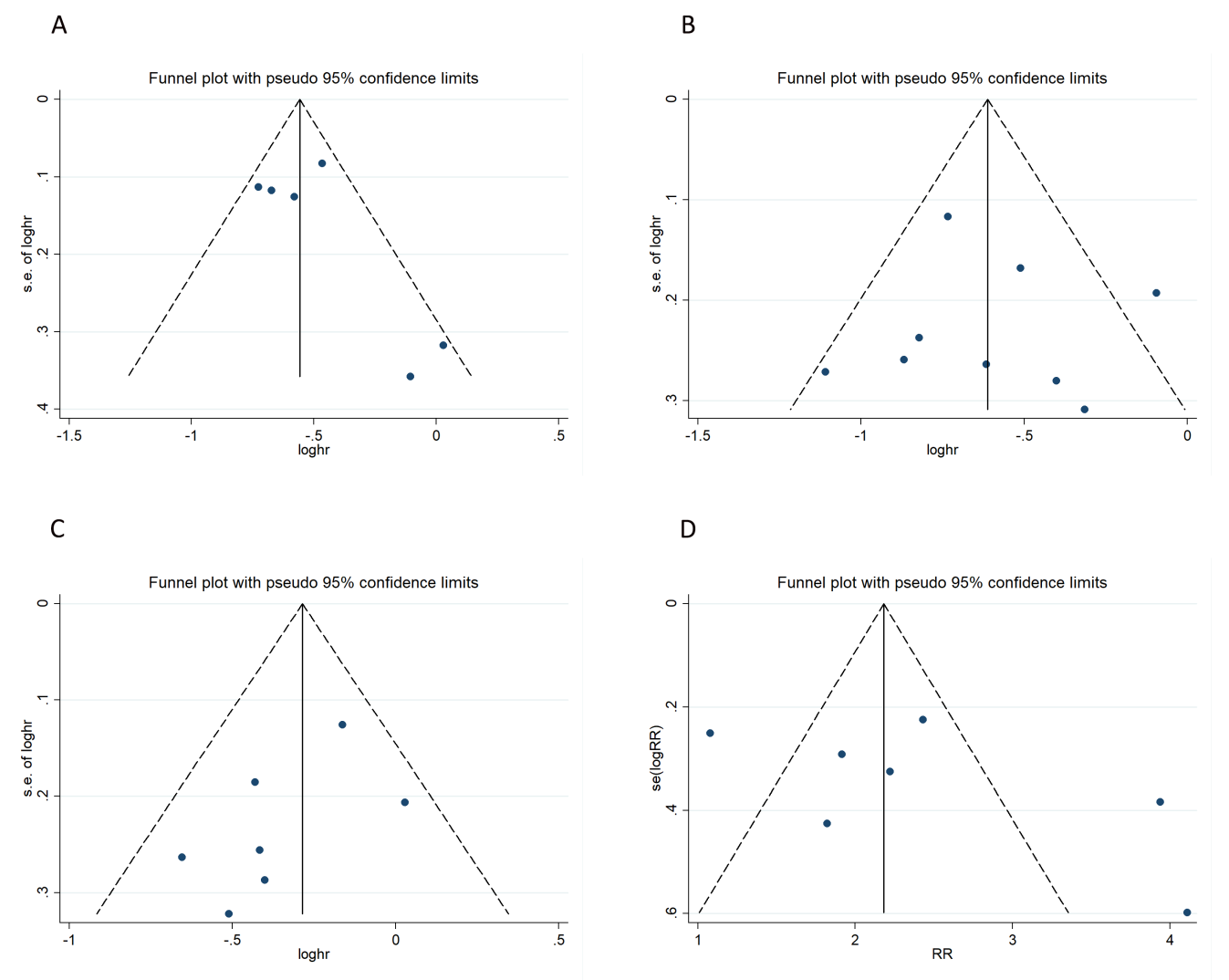

Supplement: Supplementary file 2 [file DataSheet2.docx]
