# Supplementary material for: Efficacy and safety of anti-angiogenic drugs combined with chemotherapy in the treatment of platinum-sensitive/resistant ovarian cancer: a meta-analysis with trial sequential analysis of randomized controlled trials
Source: Front Pharmacol. 2024 Nov 21;15:1446403. doi: 10.3389/fphar.2024.1446403 (PMC11617189; doi:10.3389/fphar.2024.1446403)
Supplement: Supplementary file 3 [file DataSheet1.docx]

| **PubMed 545** |
| --- |
| #1 (anti-angiogenic) OR (angiogenesis inhibitor) OR (antiangiogenetic) OR (anti-angiogenesis) OR (vascular endothelial growth factor) OR (VEGF) OR (VEGFR) OR (VEGF-R) OR (anti-VEGF) OR (Bevacizumab) OR (cediranib) OR (SU11248) OR (recentin) OR (Pazopanib) OR (AZD2171) OR (zaltrap) OR (GW786034) OR (AMG386) OR (Afibercept) OR (BIBF 1120) OR (Votrient) OR (axitinib) OR (vargatef) OR (AEE788) OR (Nintedanib) OR (BAY43-9006) OR (Avastin) OR (Sorafenib) OR (Imatinib) OR (STI571) OR (Sunitinib) OR (BAY 545-9085) OR (vandetanib) OR (Nexavar) OR (Trebananib) OR (Perifosine) OR (NSC724772) OR (AG-013736) OR (anlotinib) OR (apatinib) |
| #2 (ovar*) AND (cancer* OR tumor* OR tumour* OR carcinoma* OR neoplasm* OR malignan*) |
| #3 (randomized controlled trial) OR (RCT) OR (controlled clinical trial) OR (random*) |
| #4 #1 AND #2 AND #3 |
| **Web of Science 1509** |
| #1 TS=((anti-angiogenic) OR (angiogenesis inhibitor) OR (antiangiogenetic) OR (anti-angiogenesis) OR (vascular endothelial growth factor) OR (VEGF) OR (VEGFR) OR (VEGF-R) OR (anti-VEGF) OR (Bevacizumab) OR (cediranib) OR (SU11248) OR (recentin) OR (Pazopanib) OR (AZD2171) OR (zaltrap) OR (GW786034) OR (AMG386) OR (Afibercept) OR (BIBF 1120) OR (Votrient) OR (axitinib) OR (vargatef) OR (AEE788) OR (Nintedanib) OR (BAY43-9006) OR (Avastin) OR (Sorafenib) OR (Imatinib) OR (STI571) OR (Sunitinib) OR (BAY 545-9085) OR (vandetanib) OR (Nexavar) OR (Trebananib) OR (Perifosine) OR (NSC724772) OR (AG-013736) OR (anlotinib) OR (apatinib)) |
| #2 TS=((ovar*) AND (cancer* OR tumor* OR tumour* OR carcinoma* OR neoplasm* OR malignan*)) |
| #3 TS=((randomized controlled trial) OR (RCT) OR (controlled clinical trial) OR (random*)) |
| #4 #1 AND #2 AND #3 |
| **The Cochrane Library 1089** |
| #1 All Text=((anti-angiogenic) OR (angiogenesis inhibitor) OR (antiangiogenetic) OR (anti-angiogenesis) OR (vascular endothelial growth factor) OR (VEGF) OR (VEGFR) OR (VEGF-R) OR (anti-VEGF) OR (Bevacizumab) OR (cediranib) OR (SU11248) OR (recentin) OR (Pazopanib) OR (AZD2171) OR (zaltrap) OR (GW786034) OR (AMG386) OR (Afibercept) OR (BIBF 1120) OR (Votrient) OR (axitinib) OR (vargatef) OR (AEE788) OR (Nintedanib) OR (Avastin) OR (Sorafenib) OR (Imatinib) OR (STI571) OR (Sunitinib) OR (vandetanib) OR (Nexavar) OR (Trebananib) OR (Perifosine) OR (NSC724772) OR (AG-013736) OR (anlotinib) OR (apatinib)) |
| #2 All Text=((ovar*) AND (cancer* OR tumor* OR tumour* OR carcinoma* OR neoplasm* OR malignan*)) |
| #3 All Text=((randomized controlled trial) OR (RCT) OR (controlled clinical trial) OR (random*)) |
| #4 #1 AND #2 AND #3 |
| **Embase 1053** |
| #1 'anti angiogenic':ti,ab,kw OR 'angiogenesis inhibitor':ti,ab,kw OR antiangiogenetic:ti,ab,kw OR 'anti angiogenesis':ti,ab,kw OR 'vascular endothelial growth factor':ti,ab,kw OR vegf:ti,ab,kw OR vegfr:ti,ab,kw OR 'vegf r':ti,ab,kw OR 'anti vegf':ti,ab,kw OR bevacizumab:ti,ab,kw OR cediranib:ti,ab,kw OR su11248:ti,ab,kw OR recentin:ti,ab,kw OR pazopanib:ti,ab,kw OR azd2171:ti,ab,kw OR zaltrap:ti,ab,kw OR gw786034:ti,ab,kw OR amg386:ti,ab,kw OR afibercept:ti,ab,kw OR 'bibf 1120':ti,ab,kw OR votrient:ti,ab,kw OR axitinib:ti,ab,kw OR vargatef:ti,ab,kw OR aee788:ti,ab,kw OR nintedanib:ti,ab,kw OR 'bay43 9006':ti,ab,kw OR avastin:ti,ab,kw OR sorafenib:ti,ab,kw OR imatinib:ti,ab,kw OR sti571:ti,ab,kw OR sunitinib:ti,ab,kw OR 'bay 545-9085':ti,ab,kw OR vandetanib:ti,ab,kw OR nexavar:ti,ab,kw OR trebananib:ti,ab,kw OR perifosine:ti,ab,kw OR nsc724772:ti,ab,kw OR 'ag 013736':ti,ab,kw OR anlotinib:ti,ab,kw OR apatinib:ti,ab,kw |
| #2 ovar*:ti,ab,kw AND (cancer*:ti,ab,kw OR tumor*:ti,ab,kw OR tumour*:ti,ab,kw OR carcinoma*:ti,ab,kw OR neoplasm*:ti,ab,kw OR malignan*:ti,ab,kw) |
| #3 'randomized controlled trial':ti,ab,kw OR rct:ti,ab,kw OR 'controlled clinical trial':ti,ab,kw OR random*:ti,ab,kw |
| #4 #1 AND #2 AND #3 |
